# HyperHMM: efficient inference of evolutionary and progressive dynamics on hypercubic transition graphs

**DOI:** 10.1093/bioinformatics/btac803

**Published:** 2022-12-13

**Authors:** Marcus T Moen, Iain G Johnston

**Affiliations:** Department of Mathematics, University of Bergen, Bergen, Vestland, Norway; Department of Mathematics, University of Bergen, Bergen, Vestland, Norway; Computational Biology Unit, University of Bergen, Bergen, Vestland, Norway; CAMRIA Centre for Antimicrobial Resistance, Vestland, Norway

## Abstract

**Motivation:**

The evolution of bacterial drug resistance and other features in biology, the progression of cancer and other diseases and a wide range of broader questions can often be viewed as the sequential stochastic acquisition of binary traits (e.g. genetic changes, symptoms or characters). Using potentially noisy or incomplete data to learn the sequences by which such traits are acquired is a problem of general interest. The problem is complicated for large numbers of traits, which may, individually or synergistically, influence the probability of further acquisitions both positively and negatively. Hypercubic inference approaches, based on hidden Markov models on a hypercubic transition network, address these complications, but previous Bayesian instances can consume substantial time for converged results, limiting their practical use.

**Results:**

Here, we introduce HyperHMM, an adapted Baum–Welch (expectation–maximization) algorithm for hypercubic inference with resampling to quantify uncertainty, and show that it allows orders-of-magnitude faster inference while making few practical sacrifices compared to previous hypercubic inference approaches. We show that HyperHMM allows any combination of traits to exert arbitrary positive or negative influence on the acquisition of other traits, relaxing a common limitation of only independent trait influences. We apply this approach to synthetic and biological datasets and discuss its more general application in learning evolutionary and progressive pathways.

**Availability and implementation:**

Code for inference and visualization, and data for example cases, is freely available at https://github.com/StochasticBiology/hypercube-hmm.

**Supplementary information:**

Supplementary data are available at *Bioinformatics* online.

## 1 Introduction

Many questions in biology, medicine and beyond concern the dynamics by which a set of traits or ‘characters’ is acquired over time. These traits could be, e.g. evolving drug resistance features in pathogens, other physiological characters in evolutionary biology, mutations in cancer progression, symptoms in progressive disease, task completions in a workflow and more. Efficient ways of learning about these dynamics from available data—which may be single-time snapshots, without longitudinal tracking of individuals—can be challenging to implement.

Specific fields of study have given rise to different approaches to this question. The field of cancer evolution (Schwartz and Schäffer, 2017) has developed methods focussing on the construction of mutation graphs describing the ordering and dependence of mutational changes. Several of these approaches use a Bayesian networks picture (Beerenwinkel *et al.*, 2015; Loohuis *et al.*, 2014; Montazeri *et al.*, 2016; Ramazzotti *et al.*, 2015, 2019; Ross and Markowetz, 2016; Szabo and Boucher, 2002), which may describe dependencies between mutations as deterministic and one-way (i.e. detecting when *X* is required for *Y*, but not when *Z* has the effect of lowering the probability of *Y*). These restrictions are relaxed in approaches allowing more general influences between features. Hypercubic transition path sampling [HyperTraPS (Johnston and Williams, 2016)] and mutual hazard networks [MHN (Schill *et al.*, 2020)] both consider general (positive or negative, two-way) pairwise interactions between features (in the same form, though the inference approaches differ); the HyperTraPS picture has also been generalized further to include different structures of influence (Greenbury *et al.*, 2020), and the pairwise-interaction picture has been recently developed and accelerated (Gotovos *et al.*, 2021). Other approaches for cancer progression have been developed that use alternative methods based, for example, on the analysis of permutations (Peterson and Kovyrshina, 2017; Zhang and Wang, 2018), and Markov modelling (Hjelm *et al.*, 2006); meta-studies have compared the performance of several of these approaches (Diaz-Colunga and Diaz-Uriarte, 2021; Diaz-Uriarte and Vasallo, 2019).

In the (not disconnected) field of evolutionary biology, several approaches have been developed for describing and predicting the appearance of traits (typically called ‘characters’ in an evolutionary setting) on phylogenies. The well-known ‘Mk’ model (Lewis, 2001; Pagel, 1994) and its extensions, for example, use a Markov model picture to consider how a discrete-valued character changes on a phylogeny. In an evolutionary setting, the combined problem of inferring character evolution on a phylogeny and the phylogeny structure itself is often considered (Ronquist, 2004; Yang, 2014). Bayesian approaches for phylogenetic reconstruction (Bollback, 2006; Pagel and Meade, 2006) are often combined with Markov models for character dynamics (Ronquist, 2004), describing the different states of a character or characters and allowing stochastic transitions between those states with some rates that are model parameters to be estimated. Recent developments including generalizing the influences between evolving characters to include dependence and conditionality (Bianchini and Sánchez-Baracaldo, 2021), employing flexible hidden Markov models (HMMs) to describe character dynamics (Boyko and Beaulieu, 2021), and simulation-free approaches allowing computationally tractable treatment of problems involving ensembles of possible trees (Pasqualin *et al.*, 2017). Notably, the acquisition of resistance in HIV has been explored with a ‘mutagenic tree’ approach—akin to the mutation graphs above—combining an HMM and bootstrap resampling (Beerenwinkel and Drton, 2007). The links between the cancer and evolutionary fields have been explicitly explored by several studies picturing cancer progression as an evolutionary process (Greenbury *et al.*, 2020; Youn and Simon, 2012).

**Fig. 1.**
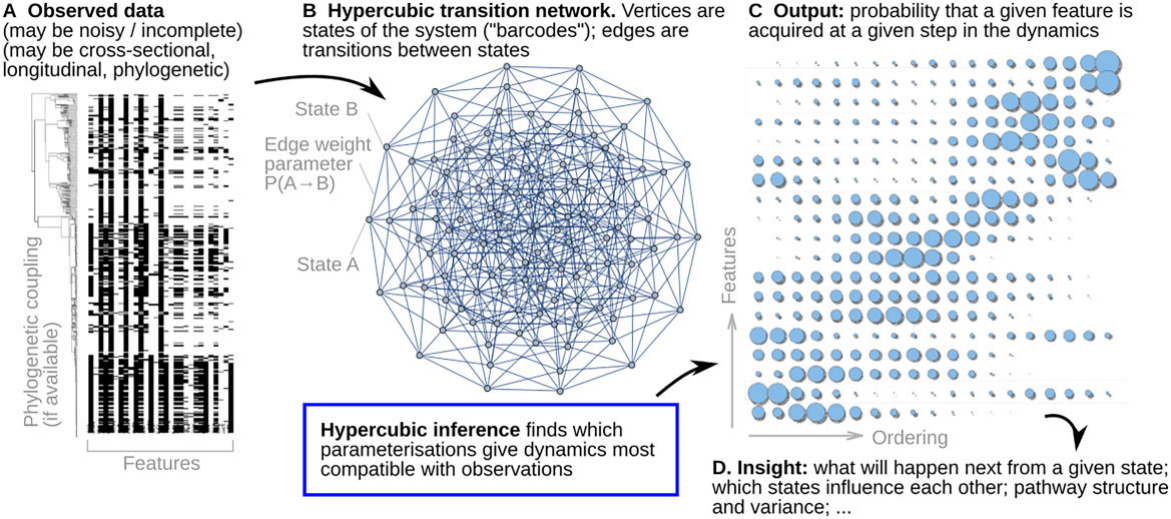
Overview of hypercubic inference. (**A**) Observed data in the form of presence/absence ‘barcodes’ for each observation, which may be incomplete or noisy, and may be independent (cross-sectional), longitudinal or phylogenetically coupled; here, TB resistance data from Casali *et al.* (2014). (**B**) The hypercubic transition network model for dynamics, where a system proceeds via a series of transitions from one vertex to another. Each vertex is a different ‘barcode’ state, edges give transition probabilities between states. Hypercubic inference learns these transition probabilities from data, finding the parameterization most compatible with a set of emitted observations. (**C**) The learned parameterization can be interpreted in several ways—as a probability map of which feature is likely acquired at which stage, explicit pathways through the hypercube space, relationships between feature orderings and more. (**D**) Scientific insight follows from interpreting these results

In parallel, several studies have considered a particular class of applied problems, which we will call ‘hypercubic inference’, applied in cancer progression (Greenbury *et al.*, 2020; Schill *et al.*, 2020), evolutionary biology (Greenbury *et al.*, 2020; Johnston and Røyrvik, 2020; Johnston and Williams, 2016) and progression of other diseases including severe malaria (Johnston *et al.*, 2019). In terms of the systems involved, this picture involves evolution progressing via the sequential, irreversible, stochastic acquisition of discrete traits [also referred to as monotonic accumulation (Ramazzotti *et al.*, 2019)]. Rather than focussing on individual traits/characters as the elements of the system, these approaches consider every possible state of a system involving *L* traits—thus, explicitly considering every combination of trait values, and thus accounting for completely general influences of any subset of current trait values and the stochastic acquisition of another. There are therefore no assumptions of deterministic or one-way relationships (Greenbury *et al.*, 2020; Schill *et al.*, 2020). The transition graph linking possible states is then a directed hypercubic graph, and edge weights (model parameters to be estimated) can be used to control the probabilities of different dynamic pathways (Fig. 1). We are concerned with the inverse problem of learning the structure of, and variability in, pathways on this hypercube from observed samples of the evolving system. The set of observations used to parameterize the hypercubic model may, in different scientific contexts, be cross-sectional, longitudinal or phylogenetically coupled (Greenbury *et al.*, 2020). The ability to account for samples linked by temporal or phylogenetic relationships, rather than only independent cross-sectional samples, is another strength of this class of approach.

HyperTraPS (Greenbury *et al.*, 2020; Johnston and Williams, 2016) and its precursor phenotypic landscape inference (Williams *et al.*, 2013) are Bayesian approaches, using likelihood estimates to build posterior distributions over the parameterizations of the hypercube. The representation of parameters is flexible, with a function used to construct each edge weight from some potentially lower-dimensional representation, including the proportional hazards picture also used in Schill *et al.* (2020). Regularization approaches have been used to seek the parameter representation most compatible with given data, which itself informs on the generative relationships between features (Greenbury *et al.*, 2020). HyperTraPS has been used to infer the evolution of efficient photosynthesis in plants (Williams *et al.*, 2013), gene loss in mitochondria (Johnston and Williams, 2016), as well as the progression of severe malaria (Johnston *et al.*, 2019), the emergence of tool use in animals (Johnston and Røyrvik, 2020) and the participation of students in digital learning (Peach *et al.*, 2021).

In addition to relaxing the common deterministic, acyclic, one-way dependencies as cited in Schill *et al.* (2020), these hypercubic inference approaches have several features, which potentially allow a more general analysis than alternative methods. First, the complete state space of all possible presence/absence combinations is considered, rather than a restricted set of pathways as in the mutagenic network picture (Beerenwinkel and Drton, 2007). Second, no restrictions are placed on how traits influence each other. The adoption of the parameterization protocol called *L*^2^ [in Johnston and Williams (2016) and Greenbury *et al.* (2020)] or mutual hazards [in Schill *et al.* (2020) and Gotovos *et al.* (2021)] means that each trait can independently influence the acquisition of another; the less restricted parameterization allowed by hypercubic inference supports (positive and negative) synergistic influence of pairs, triplets and any combinations of traits. If there is sufficient data, any state-specific transition probability can be inferred (and if there is insufficient data, model selection and uncertainty quantification approaches can be used to place bounds on such probabilities).

While general, these Bayesian approaches, which to date rely both on Markov chain Monte Carlo (MCMC) and a sampling approach to estimate likelihoods, are computationally expensive and approximate. The inclusion of prior information is natural and arguably important for low sample sizes, to avoid overfitting a small sample. However, for larger samples, we may expect lower influence of priors on the posterior. We may also wish to avoid the Bayesian paradigm outright. We can thus naturally ask if a computationally cheaper approach can provide an output akin to a maximum-likelihood estimate for hypercube parameters—as MHN (Schill *et al.*, 2020) and their extension (Gotovos *et al.*, 2021) have done for the pairwise-interaction picture. Here, we will develop and apply HyperHMM, an alternative approach for inference of dynamic pathways on directed hypercubes without restrictions on state space or trait interactions.

## 2 Materials and methods

**Hypercubic Baum–Welch algorithm.** The derivation and intuition behind the hypercubic Baum–Welch algorithm is given more fully in the Supplementary Material. Here, we simply state the essential aspects. We are concerned with estimating the probabilities $a_{i,j}=P(X_{n}=s_{j}|X_{n-1}=s_{i})$, for a stochastic process $X_{t}$ on the hypercubic graph, where each state *s* is a node and edge $s_{i}\to s_{j}$ exists iff *j* differs from *i* by exactly one feature acquisition (Fig. 1). The algorithm broadly considers the set of possible paths on the hypercube that could lead to observations being ‘emitted’ that are compatible with our observations (see Supplementary Figs S1–S3 for examples), and seeks to find transition weights that maximize the probability of these paths.

The data, we will use are a set of potentially sequential observations *O*, where $O_{r}=o_{r,0},\ldots ,o_{r,T}$, is the *r*th sequence of observation, each labelled by an observation ordering 0,…,T. Any of these observations may be absent.

The key idea is to find probabilities, $a_{i,j}$, that maximize the likelihood of seeing all of our observations. This is done by first calculating the probabilities going forward and backward in time and storing the values for each time step. The forward probability is the probability of seeing everything up to a given time *t* given our current estimate of the transition matrix, *A*, and the backward probability is the probability of seeing everything from a given time *t* until the end. The forward probability of observation *i* at time *t* is defined recursively as:

(1) $$\alpha_{t}(i)=\sum_{j}\alpha_{t-1}(j)a_{j,i},$$

being the sum of probabilities of a transition to state *i* over previous states *j*, weighted by the probability of being at *j* at time *t*−1. The recursion is bounded by $\alpha_{0}(0^{L})=1$, since our first observation will always be $0^{L}$, and $\alpha_{0}(i)=0$ for all other states *i*. The backward probabilities are similarly defined as:

(2) $$\beta_{t}(i)=\sum_{j}\beta_{t+1}(j)a_{i,j},$$

summing weighted probabilities of transitions from state *i* at time *t* to state *j* at time *t *+* *1, where the recursion now follows backwards in time bounded by $\beta_{T}(i)=1$ for all *i*, as an observation at *t *=* T* begins a backwards-time pathway.

Combining these probabilities will give us a ‘skeleton’ with all possible pathways given the observation (Supplementary Figs S1–S3). Using this, we can calculate the probability of being in any two states at times *t* and *t *+* *1 and update the transition matrix based on this information. These probabilities are called the *ξ*-probabilities and are defined as:

(3) $$\xi_{t}(i,j)=P(o_{t}=i,o_{t+1}=j|O,A)=\frac{\alpha_{t}(i)a_{i,j}\beta_{t+1}(j)}{P(O|A)},$$

where P(O|A) is the probability of seeing the given observation sequence given our current estimate of the transition matrix *A*. We then update the transition probabilities for each round according to Equation (4), which is just a normalization of the *ξ*-probabilities (see Supplementary Material for derivation):

(4) $${\hat{a}}_{i,j}=\frac{\sum_{r=1}^{R}\sum_{t=1}^{T-1}\xi_{t,r}(i,j)}{\sum_{r=1}^{R}\sum_{k=1}^{N}\sum_{t=1}^{T-1}\xi_{t,r}(i,k)}.$$

To assess convergence of the algorithm, we calculate the maximum change in any transition probability. For all of the cases in Section 2, we have used a convergence criterion of 0.001, so that if no transition probability changes by more than an absolute value of 0.001 between one iteration and the next, we terminate the algorithm.

**Bootstrap resampling.** We used 100 bootstrap resamples of observed transitions for estimating uncertainty in the HBW algorithm, and report the standard deviation of summary statistics over the set of resamples.

**HyperTraPS.** HyperTraPS was implemented following Greenbury *et al.* (2020), using $2\times 10^{3}$ samples to estimate likelihoods and a reduced parameterization mapping *L*^2^ values to the edges of the hypercubic transition network. Priors involved a uniform probability of any remaining feature being acquired at any time step. The results from HyperTraPS were obtained after preliminary investigation to find the optimal perturbation kernel for MCMC convergence. For the simple synthetic cases this was σ=0.75; for the ovarian and TB cases it was σ=0.25.

**Convergence.** For the HyperHMM approach, we recorded how long it took for the inferred parameter set to converge to a 0.001 level, with 100 bootstraps to quantify uncertainty. For HyperTraPS, we recorded how long it took for the inferred ordering posteriors to converge to a 0.001 level. We did not focus on the posteriors on individual transition parameters, because many of these are unconstrained by a given dataset and (in the absence of a sparsity prior) take a long time to recapture the prior, for negligible contribution to the inferred dynamics. In a sense this criterion allows HyperTraPS a looser definition of convergence; however, even given this laxity, the speedup from the HyperHMM approach is striking (see text).

**Summary of inferred dynamics with ordering probabilities.** To summarize HyperHMM outputs, 10^5^ random walkers are simulated on the maximum-likelihood hypercube, and the ordering of trait acquisition for each walker is recorded. Where the bootstrap is used, this process is repeated for each resampled dataset, and the standard deviation of each ordering probability over the resampled set is recorded. To summarize HyperTraPS outputs, 10^3^ random walkers are simulated on each of 10^5^ sampled hypercubes from the posterior, and the corresponding sampled trait-ordering posteriors are recorded.

**Ancestral reconstruction for TB dataset.** Here, we followed the minimum evolution approach in Greenbury *et al.* (2020), where the ancestor of two descendants was inferred to possess a trait iff both descendants also possess it. This approach assumes that the acquisition of traits is rare, so convergent acquisition is correspondingly rare; in cases where this assumption is not safe, our approach can readily be applied across an ensemble of possible phylogenies and the resulting inferred dynamics summarized accordingly.

**Implementation.** The implementation of the code is in C++; R scripts are provided for preparing data, externally running the C++ code and retrieving and plotting results. Currently, the implementation of the HyperHMM algorithm works readily for a system of at least 20 traits on a normal laptop. The implementation is made using the Compressed Row Storage format for representing sparse matrices. Code for inference and visualization is freely available at https://github.com/StochasticBiology/hypercube-hmm.

## 3 Results

### 3.1 A hypercubic Baum–Welch algorithm for efficient inference of paths on a transition hypercube

Under a hypercubic transition graph model, every possible state of the system is represented as a binary string of length *L*, where 0 and 1 at the *i*th position correspond, respectively, to absence or presence of the *i*th trait (Fig. 1). Traits are acquired stochastically and irreversibly, meaning once a trait has been acquired it cannot be lost (Greenbury *et al.*, 2020). A hypercubic transition graph is then constructed, with each node corresponding to a state, and a weighted edge from node *a* to node *b* if *b* differs from *a* by the acquisition of exactly one trait. We usually picture a given instance of the system (e.g. a patient with a progressive disease) moving over the hypercube from the binary string of all 0s towards (but not necessarily reaching) the binary string of all 1s, probabilistically following outgoing edges from a given state according to their relative weights. The goal of hypercubic inference is to learn the set(s) of edge weights that are most compatible with the observed dynamics of a given system. To this end, we consider a HMM likelihood based on emissions from processes on this transition graph (Rabiner and Juang, 1986). In the simplest case, an emission corresponds simply to the state at the current node, but an HMM approach also allows us to account for noisy and incomplete emissions.

HyperTraPS is an algorithm estimating the likelihood of a given observed transition from some state *a* to some state *b* (not necessarily only one trait apart). Given the large number of paths that can generally exist between two nodes, HyperTraPS uses biased random walkers to estimate this likelihood, which is then embedded in a Bayesian framework using MCMC for parameter estimation. The fact that this likelihood is approximate raises issues of MCMC convergence, which require corresponding algorithmic complexity to address (Greenbury *et al.*, 2020; Murray and Graham, 2016) and the Bayesian nature of the parameter search require substantial computer time.

Here, we propose HyperHMM, an alternative using (i) an adaptation of the well-known Baum–Welch algorithm (Baum *et al.*, 1970; Rabiner and Juang, 1986) for the hypercubic transition graph to estimate parameters without requiring an approximate likelihood, and (ii) a frequentist approach using resampling rather than the fully Bayesian approach for uncertainty quantification. Both (i) and (ii) allow substantial computational gains over the usual implementation of HyperTraPS, reducing runtimes from hours to seconds (see below). A similar computational paradigm has been used previously [e.g. in Beerenwinkel and Drton (2007)]—here, following HyperTraPS, it is instead applied to learn the hypercubic transition network without restrictions on parameter structure or state space. The Baum–Welch algorithm is an expectation–maximization algorithm that learns maximum-likelihood transition probabilities in an HMM from a given set of observations, and provides the maximum-likelihood counterpart to the Bayesian approach previously used with HyperTraPS (Greenbury *et al.*, 2020; Johnston and Williams, 2016). The core hypercubic Baum–Welch algorithm is given in Algorithm 1, illustrated in Figure 1 and derived in Section 4 and Supplementary Material.

Algorithm 1 The Baum–Welch algorithm for inference on hypercubic transition graphs. The algorithm proceeds by iteratively estimating forward and backward probabilities of the different transitions observed in the dataset, given a current estimate of the hypercubic transition matrix, then updating this estimate to increase the probabilities of these observations, until a convergence criterion is met. The specific form of the P(…) functions, providing the probability estimates, is given in Section 4.

Input: L= the number of traits, and $O=O_{1},\ldots ,O_{R}=$ all the *R* independent observations. Each $O_{r}=o_{r,0},\ldots ,o_{r,T}$ is a sequence of specific states, or markers denoting an unknown state, that arises from observed data (e.g. 000,001,?,? corresponds to an observation of 000→001 for an *L *=* *3 system). Output: estimated transition matrix $\hat{A}$ describing the probability of a transition between two states.

1: Select a first estimation of transition matrix $\hat{A}$. A natural choice is uniform probabilities over all outgoing edges from a given node.

2: Let $N=2^{L}$ be the number of states, and T=L+1.

3: **while** Not convergence **do**

4:  **for** *r* = 1,…,*r* = *R* **do**

5:    **for** *t* = 0,…,*t* = *T* **do**

6:     $\alpha_{t}(i)=P(o_{r,1},\ldots ,o_{r,t}=i|\hat{A})$

7:     $\beta_{t}(i)=P(o_{r,t+1},\ldots ,o_{r,T}|o_{r,t}=i,\hat{A})$

8:    **end for**

9:    **for** *t* = 0,…,*t* = *T*−1 **do**

10:     $\xi_{r,t}(i,j)=P(o_{r,t}=i,o_{r,t+1}=j|\hat{A})=\frac{\alpha_{t}(i)a_{i,j}\beta_{t}(i)}{P(O|\hat{A})}$

11:   **end for**

12:   **end for**

13:    ${\hat{a}}_{i,j}=\frac{\sum_{r}\sum_{t}\xi_{r,t}(i,j)}{\sum_{r}\sum_{k}\sum_{t}\xi_{r,t}(i,k)},\;\;\forall i,j$

14:    $\hat{A}=\left(\begin{array}{cccc} {\hat{a}}_{1,1} & {\hat{a}}_{1,2} & \cdots & {\hat{a}}_{1,N}\\ {\hat{a}}_{2,1} & {\hat{a}}_{2,2} & \cdots & {\hat{a}}_{2,N}\\ \vdots & \vdots & \ddots & \vdots \\ {\hat{a}}_{N,1} & {\hat{a}}_{N,2} & \cdots & {\hat{a}}_{N,N} \end{array}\right)$

15: **end while**

### 3.2 Synthetic test cases requiring different interaction structures between traits

We will start by looking at two synthetic cases involving simple preconstructed datasets, previously used to test HyperTraPS (Greenbury *et al.*, 2020). In the first case, we construct a dataset reflecting a single evolutionary pathway, where the acquisition of features proceeds from the first to the last indexed. We begin with this simple system with *L *=* *5 traits, with corresponding ‘data’ consisting of the observations 10000,11000,11100,11110. To demonstrate the algorithm’s ability to capture distinct pathway structures, and negative interactions between traits, we also consider a case with two competing pathways. Here, the first trait to be acquired is equally likely to be the first or last indexed. If the first, evolution proceeds as previously, but if the last, it proceeds in the ‘opposite direction’, with traits being acquired last to first in indexing order. The first step on each pathway thus represses the other pathway. 

**Fig. 2.**
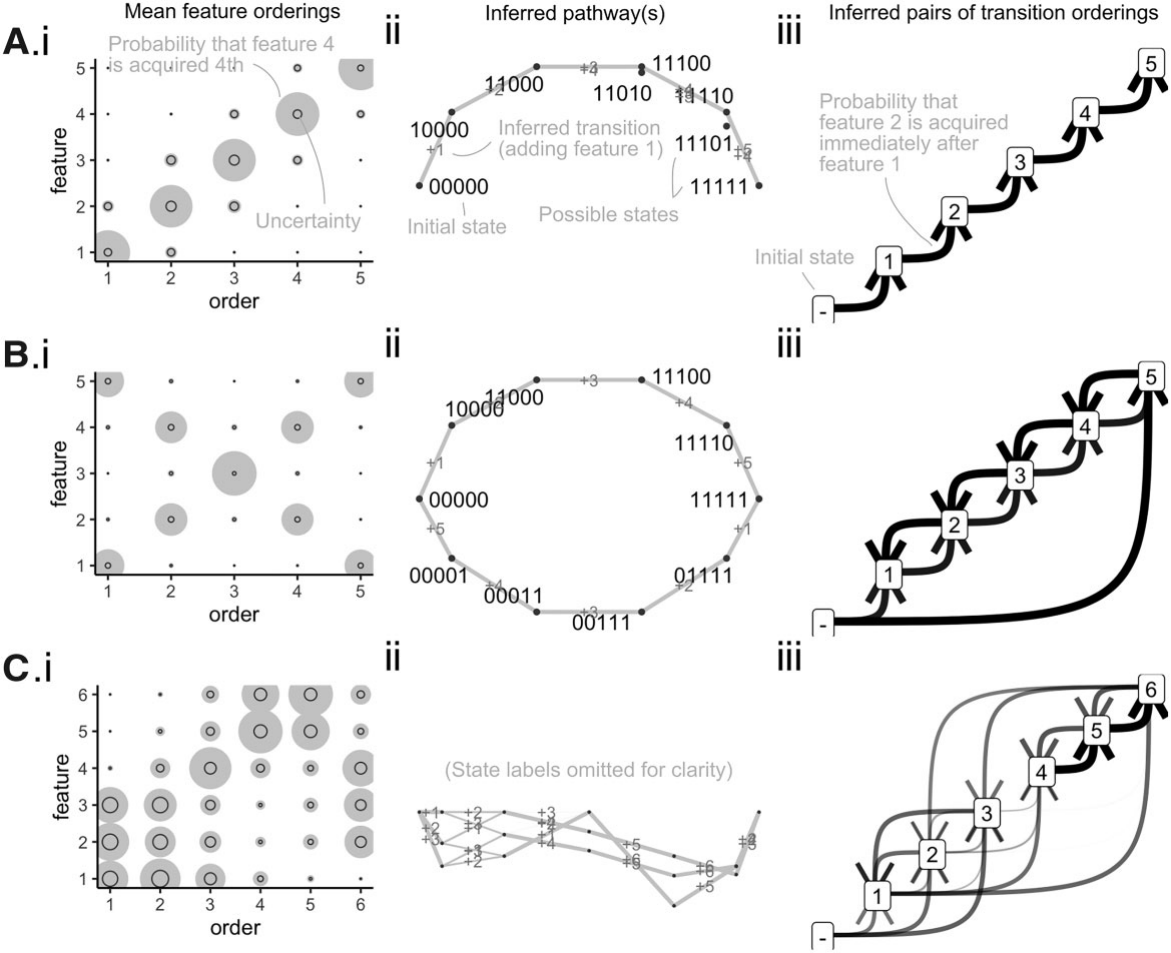
Inferred dynamics for synthetic test datasets. Synthetic examples from the text: (**A**) single pathway; (**B**) double competing pathways; and (**C**) synergistic logical interactions between traits. (i) Summary output of HyperHMM algorithm reflecting averaged trait orderings. Bubbles show the probability of acquiring trait *y* at time *x*; black circles in the HyperHMM plots shows the standard deviation after 100 bootstraps. (ii) Visualization of inferred paths on the hypercubic transition network. Individual edge labels describe which feature is changed at each transition; edge weights correspond to the probability of a given transition. (iii) Probabilistic feature graphs for orderings of features changes. An edge from *a* to *b* corresponds to acquisition of *b* directly following acquisition of *a* in inferred dynamics—gives the initial state with no features. Edge weights correspond to the frequency with which given transitions are observed in simulated dynamics

Figure 2A and B shows the results of HyperHMM inference for these two example cases. Supplementary Figure S4 expands these results to compare inference results for different sample size *N*, trait count *L*, and to compare HyperHMM and Bayesian HyperTraPS approaches. The single pathway structure is intuitively captured, with increasing certainty as the dataset size increases. The uncertainty derived from resampling with the HBW algorithm agrees well with the posteriors from HyperTraPS (Supplementary Fig. S4). HyperTraPS, as a Bayesian approach, is informed by its priors, which in this case are simply uniform acquisition probability over all options. For low *N* the influence of these priors on the posteriors is greater, and correspondingly the uncertainty quantified from HBW resampling is higher.

Supplementary Figure S5 further compares the output of other inference approaches, including Capri (Ramazzotti *et al.*, 2015) [from TRONCO (De Sano *et al.*, 2016)], Oncotree (Szabo and Pappas, 2022) (https://CRAN.R-project.org/package=Oncotree) and MHN (Schill *et al.*, 2020), for the double-pathway case. As Capri and Oncotree cannot account for mutually repressing pathways, they do not capture the full structure of this system, instead identifying a single path or putting uniform probability over many. MHN and HyperTraPS allow mutual repression, and hence capture this structure (to a very similar extent, as the target of MHN inference is the same as that of HyperTraPS).

A strength of HyperHMM is its ability to capture arbitrary influences of sets of multiple traits on the acquisition of other traits (other approaches allow individual, but not synergistic, influences). To demonstrate this ability, we consider another synthetic case consisting of *L *=* *6 traits. Initially, the final three traits have zero acquisition probability, and the first three traits have equal and independent acquisition probabilities. What happens next depends on whether Trait 1 is acquired before both Traits 2 and 3 are acquired. If Traits 1 and 2 (but not 3) or Traits 1 and 3 (but not 2) are acquired, evolution then proceeds to acquire Traits 4, 5 and 6, before acquiring the remaining 3 or 2. If both 2 and 3 are acquired before 1, evolution proceeds down the different pathway 6, 5 and 4, before acquiring the remaining 1. Succinctly, 1 AND (2 XOR 3)—states 110000 and 101000—leads to the 4–5–6 path; and 1 AND (2 AND 3)—state 111000—leads to 6–5–4. These multiple, bidirectional logic interactions cannot be precisely captured in a reduced-order system like HyperTraPS’ *L*^2^ or mutual hazards approaches (Greenbury *et al.*, 2020; Schill *et al.*, 2020). Supplementary Figure S6 shows a comparison of inference outputs for these and other approaches; while the pairwise approaches approximate the shape of the system, they include many spurious transitions and omit the full logical dependence between features (Supplementary Fig. S6C and D). As above, as the target of inference for MHN and HyperTraPS is the same *L*^2^ parameter structure, the models produce very similar approximations. By contrast, HyperHMM captures the higher-order behaviour through the corresponding explicit paths on the hypercubic space (Fig. 2C)—although neither summary of average ordering behaviour reveals this logical dependence (Fig. 2Ci and iii), it is revealed by considering the explicit hypercubic paths (Fig. 2Cii).

### 3.3 Ovarian cancer data

Following the verification of the HyperHMM approach on synthetic datasets, we next turned to a medical dataset that, while dated, has been used to test several algorithms for evolutionary inference (Greenbury *et al.*, 2020; Loohuis *et al.*, 2014; Szabo and Boucher, 2002). This dataset consists of snapshots of patterns of chromosomal aberrations in 87 ovarian cancer patients (Knutsen *et al.*, 2005). These are labelled by chromosome (1−23 and *X*), chromosomal arm (*p* or *q*) and variant type (addition + or loss −). HyperTraPS and others have used these data to test and benchmark inference approaches (Greenbury *et al.*, 2020). The questions include: do some aberrations occur systematically before others, does the presence of one aberration influence the acquisition probability of another and how distinct or separate are the different pathways by which cancer can evolve in this dataset?

**Fig. 3.**
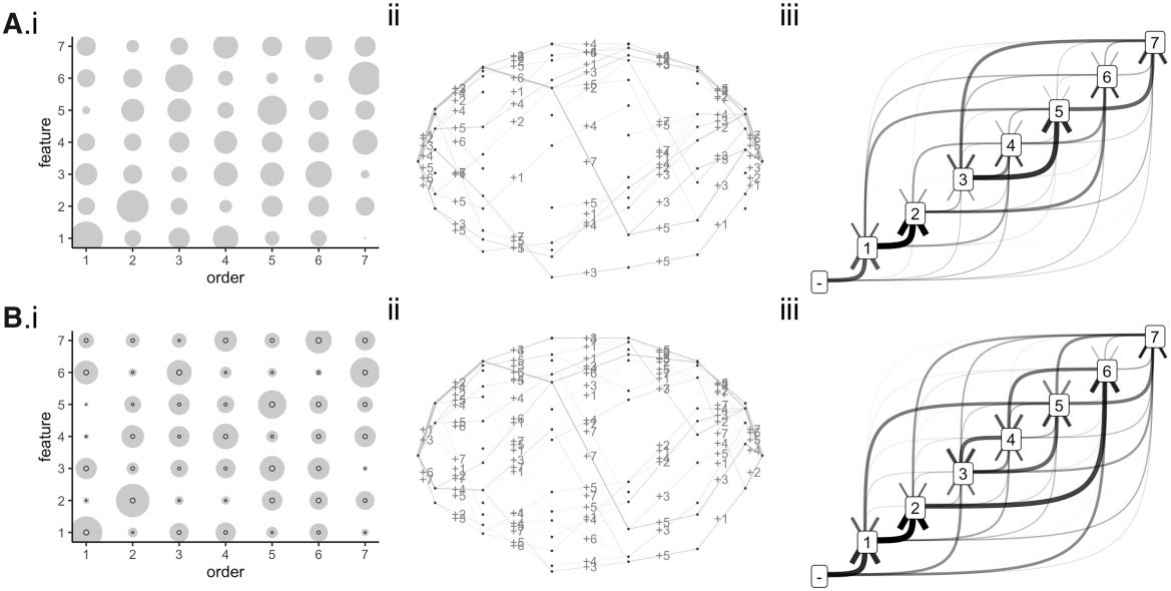
Inferred dynamics for ovarian cancer progression. Summary results using (**A**) HyperTraPS using an *L*^2^ parameterization and (**B**) HyperHMM on the ovarian cancer dataset. (i) Bubbles show the probability of getting trait *y* at time *x*; black circles in the HyperHMM plots show the standard deviation after 100 bootstraps. (ii) Visualization of inferred paths on the hypercubic transition network. Individual edge labels describe which feature is changed at each transition; edge weights correspond to the probability of a given transition. (iii) Probabilistic feature graphs for orderings of features changes. An edge from *a* to *b* corresponds to acquisition of *b* directly following acquisition of *a* in inferred dynamics; 0 gives the initial state with no features. Edge weights correspond to the frequency with which given transitions are observed in simulated dynamics. Feature labels: 1 8q+, 2 3q+, 3 5q−, 4 4q−, 5 8p−, 6 1q+ and 7 Xp−

Figure 3 shows the inferred orderings from the HyperHMM approach and from HyperTraPS. The same features are clear in both cases, with sampled hypercubic trajectories and probabilistic feature graph (Fig. 3C and D) displaying notable similarities with the HyperTraPS output found in Greenbury *et al.* (2020)—including strong weighting to the 8q+→3q+→1q+ sequence, then a more diverse range of potential dynamics thereafter. The supported trajectories here underline the ability of HyperHMM to characterize competing pathways (and negative influence of one trait on the acquisition probability of another). As discussed in Greenbury *et al.* (2020), this leads to increased flexibility in pathway identification compared to other approaches: e.g. the HyperHMM output supports acquisition of 4q− prior to 5q−, observed in 12% of samples in the data but not identified by inference approaches based on Bayesian networks (Montazeri *et al.*, 2016; Ramazzotti *et al.*, 2015). Further comparisons with other approaches are shown in Supplementary Figure S7. HyperHMM favours the same set of initial steps as Oncotree, but thereafter shares clear similarities in inferred hypercubic transitions with HyperTraPS (and MHN, which here assigns a more uniform spread of probabilities over pathways). Generally, hypercubic inference approaches allow a relaxation of the graph of trait relationships away from the tree structure enforced by many approaches to allow bidirectional interactions; and they also capture more detailed information about transitions between individual states, allowing general representations of trait dependencies.

Several examples exist in this inferred example of where higher-order interactions than simple pairwise influences (as in MHN or *L*^2^ HyperTraPS) are present. A non-exhaustive set of examples for which such higher-order interactions are important can easily be found by recording instances of P(A|∅)>max(P(A|B),P(A|C))<P(A|Band C). In other words, *B* and *C* both independently decrease the probability of *A*, but their combination re-increases its probability. Several examples of this behaviour exist in the ovarian cancer dataset. One example is the influence of 5q− and 1q+ on the probability of acquiring 8q+. The base probability of the 8q+ feature is 0.473; this is substantially reduced to $1.04\times 10^{-11}$ and 0.0485 by the individual prior acquisition of either 5q− or 1q+, respectively (the base probabilities of which are 0.196 and 0.234 respectively). However, the prior acquisition of both these traits re-elevates the probability of 8q+ to 0.132. Of course, this condition is sufficient but not necessary for higher-order influence; many other possibilities exist, but we use this as a simple example.

### 3.4 Multidrug resistance in tuberculosis

As a final test of the approach using biomedically pertinent data, we next turned to an evolutionary question—how multidrug resistance evolves in tuberculosis (TB). This global problem involves the TB bacterium acquiring resistance to the antimicrobial drugs used to treat it. We again use a dataset that has previously been used to test HyperTraPS (Greenbury *et al.*, 2020): specifically, the study of Casali *et al.* (2014). Here, drug resistance profiles of different but related strains of TB were experimentally characterized, producing barcodes of resistance/susceptibility that are connected by an estimated phylogeny.

Because of this phylogenetic relationship between samples (Fig. 1A), cross-sectional approaches that assume sample independence cannot be applied, and we use the longitudinal form of HyperHMM, where pairs of observations (ancestor–descendant) are considered as the fundamental sampling elements. Following Greenbury *et al.* (2020), we estimate the barcodes of ancestral states using maximum parsimony and hence extract the set of transitions that have occurred throughout the whole lineage. This transition set is the input for our inference algorithms.

**Fig. 4.**
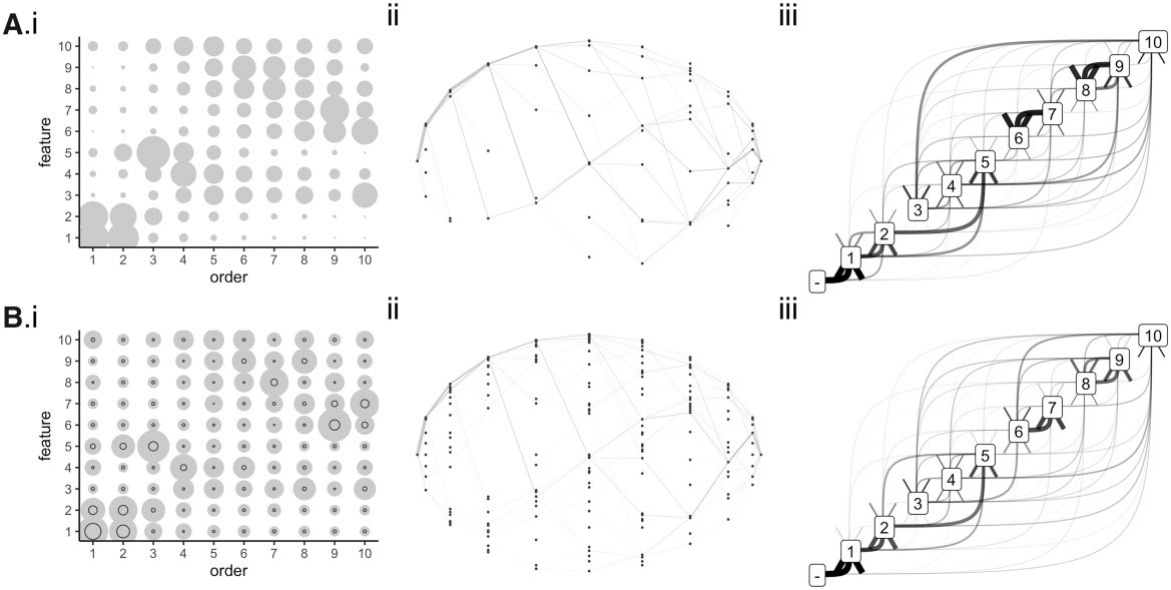
Inferred dynamics for multidrug resistance evolution in TB. Summary results using (**A**) HyperTraPS using an *L*^2^ parameterization and (**B**) HyperHMM on the TB drug resistance dataset. Each trait code is a particular drug (three-letter codes below). (i) Bubbles show the probability of getting trait *y* at time *x*; black circles in the HyperHMM plots show the standard deviation after 100 bootstraps. (ii) Visualization of inferred paths on the hypercubic transition network. Individual edge labels describe which feature is changed at each transition; edge weights correspond to the probability of a given transition. (iii) Probabilistic feature graphs for orderings of features changes. An edge from *a* to *b* corresponds to acquisition of *b* directly following acquisition of *a* in inferred dynamics; 0 gives the initial state with no features. Edge weights correspond to the frequency with which given transitions are observed in simulated dynamics. Feature labels: 1 INH, 2 RIF, 3 STR, 4 EMB, 5 PZA, 6 PRO, 7 OFL, 8 MOX, 9 CAP and 10 AMI

Once more, the structure and variability of multidrug resistance pathways is readily revealed by the HyperHMM algorithm. Again, the inferred ordering probabilities from HyperHMM agree well with the fully Bayesian approach, with some instances where HyperHMM assigns marginally increased confidence to some orderings (as above), due to the influence of the uniform priors used in HyperTraPS. Following findings in Greenbury *et al.* (2020), a strong weighting to the initial steps INH →RIF →PZA is apparent, after which the potential pathways diversify around a central ‘core’ pathway. Interestingly, this suggests that acquisition of multidrug resistance in each lineage in these samples follows one of a related set of evolutionary pathways, with some variability around later steps but the same qualitative patterning (there are not multiple, distinct, competing pathways in Fig. 4C to the extent seen in Fig. 2B). This raises the possibility of predicting the next evolutionary step for a strain with a given drug resistance profile, with potential applications to optimal treatment design [akin to Johnston *et al.* (2019) and Diaz-Colunga and Diaz-Uriarte (2021)].

The TB dataset also contains several examples where higher-order interactions are inferred. One striking example is P(AMI|∅)=0.0778; P(AMI|INH)=0.0382; $P(\mathrm{AMI}|\mathrm{PZA})<10^{-90}$; P(AMI|INH+PZA)=0.0687 (where P(INH|∅)=0.762 and P(PZA|∅)=0.0136). In other words, the probability of acquiring resistance to AMI is decreased by the acquisition of either INH or PZA independently, but their combination re-elevates the probability of acquiring AMI almost to its original level.

### 3.5 Comparison to Bayesian approach: benchmarking performance, prior information and model selection

Throughout the above investigations, we observed that the hypercubic Baum–Welch algorithm yielded results much more quickly than the HyperTraPS approach—intuitively, as a maximum-likelihood approach will typically involve less computation than Bayesian inference attempting to summarize the full parameter space. To quantify the difference in speeds, we considered simple synthetic test cases of the single- and double-pathway systems introduced above with *L *=* *5, 7 and 9, along with the real-world datasets (see Section 4). HyperHMM typically converges 2–3 orders-of-magnitude more quickly than the Bayesian implementation of HyperTraPS, without requiring preliminary or parallel tuning of the MCMC parameters (Supplementary Table S1). Quantitative details on the convergence time overall and per iteration with number of features *L* are given in Supplementary Figure S8. In Supplementary Figures S5–S7, we show comparisons with other approaches from TRONCO (De Sano *et al.*, 2016), Oncotree (Szabo and Pappas, 2022) and MHN (Schill *et al.*, 2020). Implementation issues made it hard to precisely benchmark the runtimes of each of these approaches on the same machine, but we generally observed that Capri and Oncotree ran very quickly, with MHN taking longer (on the order of seconds with our trial datasets), but using less time than HyperHMM—as expected, as the associated parameter space is smaller.

One sacrifice in going from the fully Bayesian HyperTraPS is a reduced ability to include prior information in the inference process (Greenbury *et al.*, 2020). However, this can be partly addressed within HyperHMM by changing the structure of the hypercubic transition network that underlies our model—which also allows us to perform model selection and regularization within HyperHMM. In the Supplementary Material, we outline how edge removal can capture prior information and different model structures within HyperHMM, and how simple information-based comparison statistics like the Akaike Information Criterion can be used to select an appropriate model structure for a given dataset.

## 4 Discussion

We have shown that a hypercubic Baum–Welch algorithm is an efficient alternative for inferring dynamic pathways on hypercubic transition graphs with arbitrary, potentially high-order interactions between features. Samples linked by a longitudinal or phylogenetic relationship (as in the TB case) can readily be used in the inference process, generalizing beyond independent cross-sectional samples. Arbitrary (not just pairwise) influences of sets of features on acquisition probabilities can be captured. The considerable speedup afforded over the original HyperTraPS implementation increases the set of problems that can be addressed; at the same time, no case-specific choices about perturbation kernels and likelihood estimation parameters need to be made. This simplification and speedup does not substantially challenge the approach’s ability to capture and quantify uncertainty, making it a competitive choice for inferring the structure of, and variability in, dynamic pathways—and for predicting future behaviour given current states (Diaz-Colunga and Diaz-Uriarte, 2021). In particular, HyperHMM directly gives predictions about the next step(s) that will occur from any specific state, providing potentially actionable information—e.g. to which drug a given bacterial strain will likely next evolve resistance, helping to choose a safer treatment alternative.

One advantage of the likelihood estimation process here is that noisy and incomplete observations can very easily be included in the source data, via appropriate choices for emission probabilities in the algorithm. Accounting for noisy observations is achieved by assigning each state a non-zero probability of emitting a signal that differs from its signature. The choice of this probability can be informed by the particular system, and could involve, e.g. a constant error probability *ϵ* per bit, so that the probability of emitting a signal that differs by *l* bits from the current state is *ϵ^l^* (appropriately normalized). Incomplete observations can be modelled by assigning each state compatible with a signal an equal probability of emitting that signal (e.g. 100 and 110 may emit 1?0 with equal probability). This emission probability does not itself influence the probability of those states arising in the dynamics, which is determined by the inferred parameterization.

The hypercubic Baum–Welch algorithm considers every transition edge on the hypercube—allowing arbitrary interactions between traits, as in 2C. In the examples above, because of the dramatic increase in computational efficiency, we have not coarse-grained the associated parameter space in any way. Examples up to around *L *=* *24 traits can readily be analysed on a modern computer, with that value requiring about 4 GB of memory during processing; higher trait numbers will require more memory. However, faced with larger problems, the same strategies for dimensionality reduction that were employed with HyperTraPS (Greenbury *et al.*, 2020)—and have been employed in similar hypercubic models (Schill *et al.*, 2020) could readily be employed here. This would involve a potentially simple function in Algorithm 1 mapping a reduced parameter set θ′, e.g. involving a proportional hazard mapping as in Greenbury *et al.* (2020) and Schill *et al.* (2020) to transition edge weights, and its inverse being used in the parameter update step each iteration.

It should be noted that technically the uncertainty obtained here from bootstrap resampling can be viewed as a measure of the uncertainty of the outcome of the algorithm rather than directly as a measure of the uncertainty of the parameter estimation, and that this difference can be considerable in some cases, particularly for non-identifiable parameters (Fröhlich *et al.*, 2014). In most cases, there will be edge weights in the hypercubic network that cannot be precisely estimated given a dataset (e.g. those that fall far from any likely paths generating the observations), but the contribution of these parameters to the summary behaviour of an inferred model is by nature very small. Nonetheless, uncertainty analysis without resampling, e.g. via automatic or numerical calculation of likelihood derivatives and Fisher information, would be an appropriate future extension for this approach.

Comparisons with alternative approaches for inferring dynamics in a similar way to the hypercubic picture have been shown in Johnston and Williams (2016) and Greenbury *et al.* (2020), and in Supplementary Figures S5–S7 here. Classes of approach for this problem broadly include simple logistic regression, approaches based on Bayesian networks, approaches for modelling and learning stochastic processes on phylogenies and approaches harnessing topology and/or dimensionality reduction before performing inference (Greenbury *et al.*, 2020). Previous comparisons have demonstrated that HyperTraPS typically has advantages of scaling and generality over several other approaches. Of particular note are the early method of Hjelm *et al.* (2006), where a Markov chain picture is used to construct networks describing longitudinal observations with pairwise trait interactions; Beerenwinkel and Drton (2007), who use a HMM with bootstrapping to infer mutagenic tree representations of resistance dynamics in HIV (but do not consider the dynamics through an unrestricted space of presence/absence sets); and Schill *et al.* (2020), who apply a different set of inference approaches to a very similar hypercubic model setup, while using the mutual hazards parameterization (corresponding to individual, but not pairwise or above, influences of trait on each other’s acquisition)—extended and accelerated in Gotovos *et al.* (2021). We believe that hypercubic inference approaches (HyperHMM and HyperTraPS) complement these approaches by relaxing assumptions on both the state space considered and the influences between traits, generalizing both aspects of the model (while being restricted to learning orderings, rather than continuous timings). Our aim here is to demonstrate that the HyperHMM matches HyperTraPS output—and hence retains these and other advantages—while allowing a considerable speedup that will render more large-scale problems computationally tractable.

## Supplementary Material

btac803_Supplementary_Data
