# Supplementary material for: HyperHMM: efficient inference of evolutionary and progressive dynamics on hypercubic transition graphs
Source: Bioinformatics. 2022 Dec 13;39(1):btac803. doi: 10.1093/bioinformatics/btac803 (PMC9848056; doi:10.1093/bioinformatics/btac803)
Supplement: btac803_Supplementary_Data [file btac803_supplementary_data.pdf]

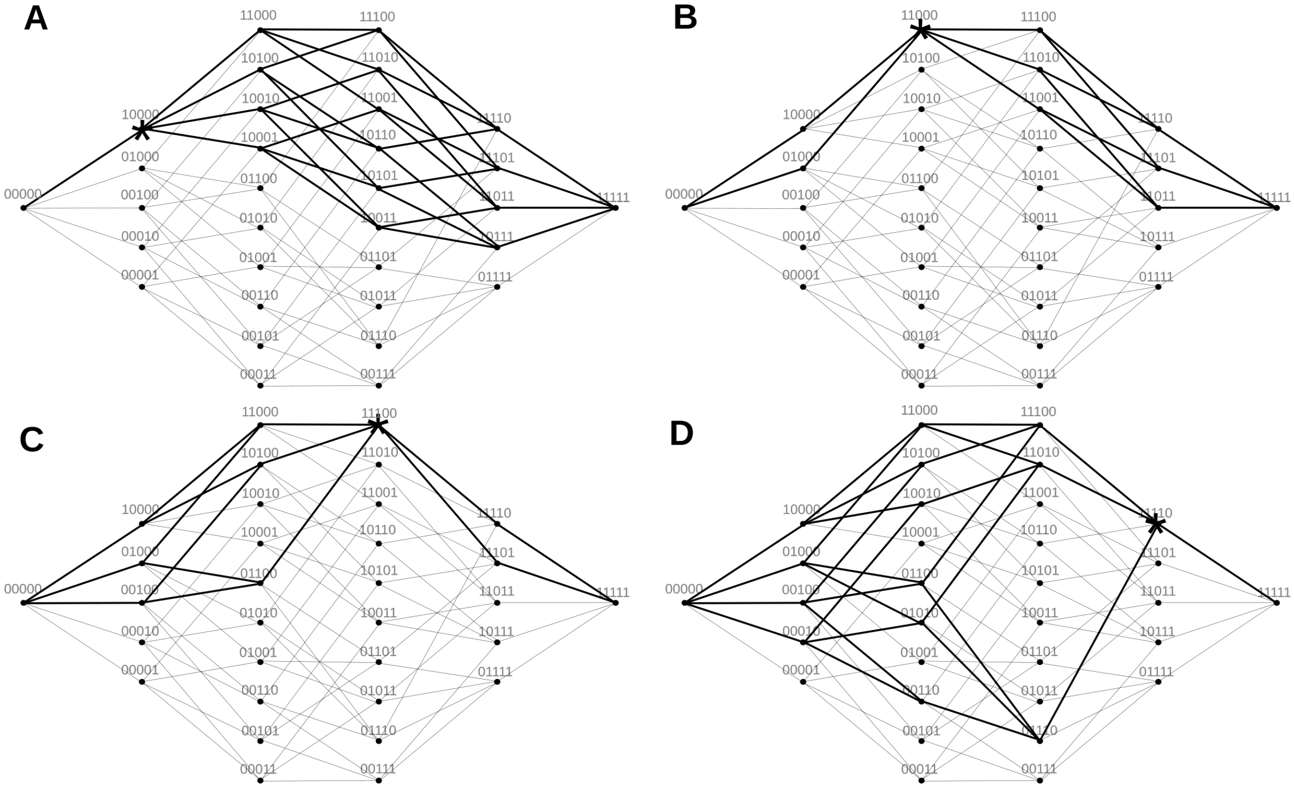


Figure S1: Illustration of hypercubic trajectories associated with an observation. Visualisation of all possible pathways given an observation on a 5D hypercube, where the black lines shows the possible pathways given the observations (marked with stars) (A) 10000, (B) 11000, (C) 11100, (D) 11110.

# Supplementary Information

## .1 Model setup

**Definition .1** (State space and observation sequences)**.** The **state space** is a set consisting of all the possible states in which the system can exist. An **observation sequence** is a time ordered sequence of observed states from a given state space, denoted as $O$.

**Definition .2** (Transition probabilities and transition probability matrix [1])**.** A **transition probability** is the probability of going from one state to another in a stochastic process. Let $\{X_t, t \in T\}$ be a stochastic process with state space $S = s_1, s_2, ..., s_N$, then the corresponding transition probabilities are $P(X_n = s_j | X_{n-1} = s_i) = a_{i,j}$, each of which is the probability of going from state $s_i$ to state $s_j$. Note that $\sum_{j=1}^{N} a_{i,j} = 1$, since we have to go to one of the states, and hence the probability of going from state $i$ to any other state has to be 1.

The **transition probability matrix**, or just the transition matrix, is the matrix representation of all the transition probabilities:

$$A = \begin{pmatrix} a_{1,1} & a_{1,2} & \cdots & a_{1.N} \\ a_{2,1} & a_{2,2} & \cdots & a_{2,N} \\ \vdots & \vdots & \ddots & \vdots \\ a_{N,1} & a_{N,2} & \cdots & a_{N,N} \end{pmatrix}$$

**Definition .3** (Emission probabilities and emission matrix [2])**.** Let $\{X_t, t \in T\}$ be a stochastic process with state space $S$, and $O$ be the sequence of observations drawn from the set $\{y_1, ..., y_m\}$. Then $b_i(y_k) = P(y_k | X_n = s_i)$ denotes the **emission probabilities** (or observation likelihoods). This is the probability of generating observation $y_k$ given that we are in state $s_i$. Note that $\sum_{k=1}^{m} b_i(y_k) = 1$.

The **emission matrix** is the matrix representation of all the emission probabilities:

$$B = \begin{pmatrix} b_1(y_1) & b_1(y_2) & \cdots & b_1(y_M) \\ b_2(y_1) & b_2(y_2) & \cdots & b_2(y_M) \\ \vdots & \vdots & \ddots & \vdots \\ b_N(y_1) & b_N(y_2) & \cdots & b_N(y_M) \end{pmatrix}$$

## .2 The Baum-Welch algorithm

A hidden Markov model (HMM) is a model wherein a system evolves according to an unobservable Markov chain, but emits signals that are observable. A given HMM is described by the $A$ and $B$ matrices above. The Baum-Welch algorithm, developed in the late 1960's and early 1970's, is a variant of the expectation-maximisation algorithm that estimates transition- and emission probabilities in a hidden Markov model, with very diverse use across fields including speech recognition, computational biology, computer vision and econometric [3].

To use the Baum-Welch algorithm one need a finite number of states and a sequence, or multiple sequences, of observations. The goal of the Baum-Welch algorithm is to use this information to estimate the transition probabilities in the underlying Markov chain, and the emission probabilities connected to the Markov chain. One important feature of the Baum-Welch algorithm is that it is only guaranteed to find a local optimum, not necessarily the global optimum, as is the case with many learning algorithms [3]. This means that we are not guaranteed the best solution every time, and that the result might depend on the initialisation of the algorithm.

We proceed by reviewing the original Baum-Welch algorithm, before describing the adaptations to the multiple signals, hypercubic case. At the core of the algorithm are several sub-functions, which are called $\alpha$-, $\beta$-, $\xi$-, and $\gamma$-function, also referred to as $\alpha$-, $\beta$-, $\xi$-, and $\gamma$-probabilities [4]. We write $\lambda = A, B$ for the set of parameters describing a particular HMM, and $q_t$ for the state of the (unobservable) HMM at time $t$.

**Definition .4** ($\alpha$-probability)**.** The **$\alpha$-probability** is the probability of seeing a given set of observations up to time $t$ and that we are in state $i$ at time $t$ given all of the hidden Markov model parameters $\lambda$:

$$\alpha_t(i) = P(o_0, ..., o_t, q_t = i|\lambda)$$

**Definition .5** ($\beta$-probability)**.** Given a state $i$ at time $t$ and the model parameters $\lambda$, the **$\beta$-probability** is the probability of observing all the observations starting from $o_{t+1}$, the observation at time $t+1$, and going to $o_T$, the observation at $T$:

$$\beta_t(i) = P(o_{t+1}, ..., o_T|q_t = i, \lambda)$$

**Definition .6** ($\xi$-probability)**.** Given an observation sequence $O = o_0, ..., o_T$ and the model parameters, the **$\xi$-probability** is the probability of being in state $i$ at time $t$ and state $j$ at time $t+1$:

$$\xi_t(i, j) = P(q_t = i, q_{t+1} = j|O, \lambda)$$

**Definition .7** ($\gamma$-probability)**.** Given an observation sequence $O = o_0, ..., o_T$ and the model parameters, the **$\gamma$-probability** is the probability of being in state $i$ at time $t$

$$\gamma_t(i) = P(q_t = i|O, \lambda)$$

The $\xi$-function and $\gamma$-function is built up using the $\alpha$-function and $\beta$-function, while the updating of transition and emission probabilities are done by using the $\xi$-function and $\gamma$-function.

The main idea of the Baum-Welch algorithm is to calculate the probability of seeing the observations when moving forward in time and when moving backward in time independently using the initial estimate of $A$ and $B$. Then these calculations can be used to find a value for being at any given state at a given time. We can then normalize these values to find the new estimate of $A$ and $B$. Iterating this process repeatedly until convergence is the Baum-Welch algorithm, given in Algorithm 1, with each step explained in more detail in Section .4.

## .3 Multiple Sequence Baum-Welch Algorithm

The original Baum-Welch algorithm takes one observation sequence, $O = o_0, ..., o_T$. In hypercubic inference we typically have a dataset involving multiple independent evolutionary or progressive instances of a system, and are therefore interested in estimating parameters that best describe the dynamics of this ensemble of instances. We therefore require a multiple-sequence adaptation of the Baum-Welch algorithm.

**Algorithm 1:** The Baum-Welch algorithm [2]. Takes a set of $T+1$ observations $o_0, ..., o_T$, estimates transition matrix $A$ and emission matrix $B$.

Select a first estimation (could be randomly) $\hat{A}$ and $\hat{B}$.
Set $\alpha_1(j) = \frac{1}{N}, j = 1, ..., N$;
Set $\beta_T(i) = 1, i = 1, ..., N$;
**while** *Not convergence* **do**

Estimate $\alpha$. $\alpha_t(j) = \sum_{i=1}^{N} \alpha_{t-1}(i)\hat{a}_{i,j}\hat{b}_j(o_t), \;\; j = 1, ..., N, \; t = 2, ..., T$;

Estimate $\beta$. $\beta_t(i) = \sum_{j=1}^{N} \beta_{t+1}(j)\hat{a}_{i,j}\hat{b}_i(o_{t+1}), \;\; i = 1, ..., N, \; t = 1, ..., T-1$

Estimate $\xi$. $\xi_t(i,j) = \frac{\alpha_t(i)a_{i,j}b_j(o_{t+1})\beta_{t+1}(j)}{\sum_{j=1}^{N}\alpha_t(j)\beta_t(j)}, \;\; i,j = 1, ..., N, \; t = 1, ..., T-1$ Estimate $\gamma$.

$\gamma_t(i) = \frac{\alpha_t(i)\beta_t(i)}{\sum_{j=1}^{N}\alpha_t(j)\beta_t(j)}, \;\; i = 1, ..., N, \; t = 1, ..., T$

Estimate $A$ and $B$ matrices. $\hat{a}_{i,j} = \frac{\sum_t \xi_t(i,j)}{\sum_k \sum_t \xi_t(i,k)}, \;\; i,j = 1, ..., N$

$\hat{b}_i(y_k) = \frac{\sum_{t=1}^{T} 1\{o_t = y_k\}\gamma_t(i)}{\sum_{t=1}^{T}\gamma_t(i)}, \;\; i = 1, ..., N, k = 1, ..., M$

$$\hat{A} = \begin{pmatrix} \hat{a}_{1,1} & \hat{a}_{1,2} & \cdots & \hat{a}_{1,N} \\ \hat{a}_{2,1} & \hat{a}_{2,2} & \cdots & \hat{a}_{2,N} \\ \vdots & \vdots & \ddots & \vdots \\ \hat{a}_{N,1} & \hat{a}_{N,2} & \cdots & \hat{a}_{N,N} \end{pmatrix}$$

$$\hat{B} = \begin{pmatrix} \hat{b}_1(y_1) & \hat{b}_1(y_2) & \cdots & \hat{b}_1(y_M) \\ \hat{b}_2(y_1) & \hat{b}_2(y_2) & \cdots & \hat{b}_2(y_M) \\ \vdots & \vdots & \ddots & \vdots \\ \hat{b}_N(y_1) & \hat{b}_N(y_2) & \cdots & \hat{b}_N(y_M) \end{pmatrix}$$

**end**

In the single-sequence Baum-Welch we update the transition probabilities using the following formula:

$$\hat{a}_{i,j} = \frac{\sum_t \xi_t(i,j)}{\sum_k \sum_t \xi_t(i,k)}, \quad i,j = 1,...,N, \tag{1}$$

estimating the probability, summed over all times, of being in $j$ the timestep after being in $i$, normalised by the total probability of being in any state $k$ the timestep after being in $i$.

Let us use the following notation, $O_r = o_{r,0},...,o_{r,T}$, to denote the $r$'th observation sequence, and $\xi_{r,t}$ is the $\xi$-probability calculated using $O_r$ at time $t$. Note that all $O_r$ is independent of each other. Then we can update the transition probabilities like this instead:

$$\hat{a}_{i,j} = \frac{\sum_r \sum_t \xi_{r,t}(i,j)}{\sum_r \sum_k \sum_t \xi_{r,t}(i,k)}, \quad i,j = 1,...,N \tag{2}$$

Which is the same as before, except that we now sum over all the independent observation sequences as well.

## .4 Hypercubic Multiple Sequence Baum-Welch Algorithm

Our main objective is to infer transition probabilities over a directed hypercube given some data. The weight of the edges on the hypercube is the transition probabilities from node $i$ to node $j$. We assume that the Markov condition holds – that is, the probability of going from one state to another is only dependent on the previous state. This allows the presence and absence of different traits to have arbitrary influence on the acquisition of further traits, but means that the history through which the existing traits were acquired does not influence future behaviour. This is intuitively appropriate for many biological situations where, for example, the fitness of an organism is a function of the organism itself and not its evolutionary history.

The state space in this case is the set of all nodes on the hypercube, meaning if we have $L$ traits there are $2^L$ possible states to be in. With a complete transition graph this would imply an extremely large transition matrix for high $L$, with $2^L \cdot 2^L$ values. However, since we are assuming a hypercubic structure – that is, that no entity can remove a trait once they have it, and that we can only get one trait at a time – this transition matrix is sparser. The number of allowed transitions are the number of edges on the hypercube, $2^{L-1}L$, which means we will have $2^{2L} - 2^{L-1}L$ zero entries in the matrix.

The observation data we are considering will typically involve transitions between two states. This will either be the transition from the $0^L$ initial state to a given observation (in the case of cross-sectional data) or the transition from some precursor state to some subsequent state (in the case of longitudinal or phylogenetic data).

We begin by picturing each independent observed transition as a subset of a longer trajectory from the node of all zeroes to the node of all ones. As steps through our hypercubic state space involve the acquisition of one trait at a time, each such long trajectory will involve $L$ steps. We use the wildcard character '?' to represent all unspecified states in a given trajectory. For example, a cross-sectional observation of 1001 would give the trajectory $0000 \rightarrow ? \rightarrow 1001 \rightarrow ? \rightarrow 1111$, and a longitudinal observation $1100 \rightarrow 1101$ would give $0000 \rightarrow ? \rightarrow 1100 \rightarrow 1101 \rightarrow 1111$.

Writing the observation set in this way gives us some advantages. First, notice that $o_0$ always equals all 0's and $o_T$ always equals all 1's, and that every observation sequence, once we have added the unknown states, are of the same length. The question mark will from now on represent all possible states to be in at that time given the observation sequence.

Let us start by looking at how we can calculate the $\alpha$-probabilities. In the normal Baum-Welch algorithm the $\alpha$-probabilities is defined as follow, $\alpha_t(i) = P(o_0,...,o_t,q_t = i|\lambda)$. Since $o_t$ is just a given state at time $t$, this gives $\alpha_t(i) = P(o_0,...,o_t = i|\lambda)$. At $t = 0$ we have $\alpha_0(i) = P(o_0 = i|\lambda) = 1$ for $i = 0^L$ (the initial state), and 0 for all other states.

For $t > 0$, we know that $\alpha_t(i) = P(o_0,...,o_t = i)$. Marginalising on the final step, it follows that $P(o_0,...,o_t = i) = \sum_j P(o_0,...,o_{t-1} = j) \cdot P(j \rightarrow i)$. Here $P(o_0,...,o_{t-1} = j) = \alpha_{t-1}(j)$ and $P(j \rightarrow i) = a_{j,i}$. This gives the following recursive formula for calculating $\alpha_t(i)$ when $t > 0$:

$$\alpha_t(i) = \sum_j \alpha_{t-1}(j) a_{j,i} \tag{3}$$

In an unrestricted case, calculating and summing this value for every state would become intractable for large $L$. The hypercubic structure of the problem dramatically simplifies the calculation. Let us first look at the sum over all states $j$. Here, $a_{j,i} = 0$ for every state $j$ where it is not possible to move from $j$ to $i$. This

means that we only need to sum over all states where it is possible to move from $j$ to $i$. Let us define this set as follows:

**Definition .8.** $j \in D_i$ if, and only if, $a_{j,i} > 0$

If we know the state at time $t$, we only need to calculate $\alpha_t(i)$ for that given state and set all other $\alpha_t$ values to 0. However, if we do not know the state at time $t$, we need to calculate $\alpha_t(i)$ for every $i$ accessible from $j$ where $\alpha_{t-1}(j) > 0$. This set of accessible $i$ states will be defined as $F_t$ as follows:

**Definition .9.** $i \in F_t$ if and only if there exists a state $j$ such that $a_{j,i} > 0$ and $\alpha_{t-1}(j) > 0$.

This gives us the following expression for $\alpha_t(i)$.

**Definition .10.** [$\alpha$-probabilities]
$$\alpha_t(i) = \begin{cases} \sum_{j \in D_i} \alpha_{t-1}(j) a_{j,i} & \text{if } i \in F_t \\ 0, & \text{otherwise} \end{cases}$$

This calculation only applies to the case where $o_t$ is unknown. If $o_t$, is known we would know that every $\alpha_t(i) = 0$ as long as $i \neq o_t$. The calculation for $\alpha_t(o_t)$ is however the same as the upper equation in Def. .10.

The $\beta$-probabilities are calculated in a similar way. We start by looking at the most simple case, when $t = T$. Since we do not have any observations after time $T$, we will define $\beta_T(i)$ to be 1 for every $i \in S$.

For $t < T$ we have the following expression in the normal Baum-Welch algorithm: $\beta_t(i) = P(o_{t+1}, ..., o_T | o_t = i, \lambda)$. This equation can be rewritten such that we gain a recursive formula, like in Def. .10.

$$\begin{aligned} \beta_t(i) &= P(o_{t+1}, ..., o_T | o_t = i, \lambda) \\ &= \sum_j P(o_{t+2}, ..., o_T | o_{t+1} = j, \lambda) \cdot a_{i, o_{t+1} = j} \\ &= \sum_j \beta_{t+1}(j) \cdot a_{i,j} \end{aligned}$$

Like with the $\alpha$-probabilities we do not need to calculate everything all the time, but the general rule for calculating the $\beta$-probabilities is:

**Definition .11.** Let $B_t$ denote the set of all possible states to be in at time $t$. The set $B_t$ hence contains all binary strings which has $t$ 1's.

**Definition .12.** [$\beta$-probabilities] $\beta_t(i) = \begin{cases} \sum_{j \in B_{t+1}} \beta_{t+1}(j) a_{i,j}, & \text{if } i \in B_t \\ 0, & \text{otherwise} \end{cases}$, for $T > t$

For the $\xi$-probabilities we have to do something slightly different. The most simple case is when both $o_t$ and $o_{t+1}$ are known. When this is the case, we have full information and know that $\xi_t(i,j) = 1$ when $i = o_t$ and $j = o_{t+1}$. For every other value of $i$ and $j$, $\xi_t(i,j) = 0$.

In general, $\xi_t(i,j)$ can be calculated using the $\alpha$- and $\beta$-probabilities. We know the probability of seeing everything up to time $t$, and being in state $i$ at time $t$ ($\alpha$-probabilities), we know the probability of going from $i$ to $j$ ($a_{i,j}$), and we know the probability of seeing everything after time $t$ given state $j$ ($\beta$-probabilities). Then $\xi_t(i,j)$ will just be all these probabilities multiplied together.

As with the $\alpha$- and $\beta$-probabilities we can use the known observation sequence to cut down the number of needed calculations. When $o_t$ is known and $o_{t+1}$ is unknown, we only need to calculate for every $j$ which is possible to reach from $i$. If the opposite is true, that we know $o_{t+1}$ but not $o_t$, we only need to calculate for every $i$ where it is possible to go to $j$.

The last possible case is when none of the states are known. In this case we need to calculate $\xi_t(i,j)$ for every state $i$ which is possible to be in at time $t$ and every state it is possible to go to from that state.

**Definition .13** ($\xi$-probabilities)**.** For the $\xi$-probabilities we will have these four cases:

If both $o_t$ and $o_{t+1}$ are known:
$$\xi_t(i,j) = \begin{cases} 1, & \text{if } i = o_t \text{ and } j = o_{t+1} \\ 0, & \text{otherwise} \end{cases}$$

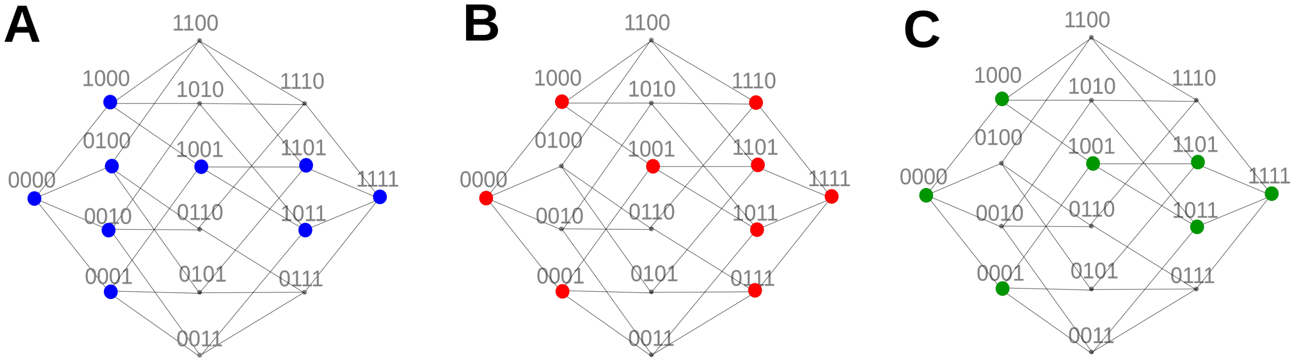


Figure S2: **Example nodes considered in the HBW calculation.** Given the observation 1001, (A) shows nodes that will have $\alpha_t(i) > 0$ for any time $t$; (B) shows nodes that will have $\beta_t(i) > 0$ for any time $t$; (C) shows nodes that are present in both (A) and (B). All the edges connecting two nodes here will be given some weight when uploading the transition probabilities.

If only $o_t$ is known:

$$\xi_t(i,j) = \begin{cases} \frac{\alpha_t(i)a_{i,j}\beta_{t+1}(j)}{P(O|\lambda)}, & \text{if } j \in D_i \text{ and } i = o_t \\ 0, & \text{otherwise} \end{cases}$$

If only $o_{t+1}$ is known:

$$\xi_t(i,j) = \begin{cases} \frac{\alpha_t(i)a_{i,j}\beta_{t+1}(j)}{P(O|\lambda)}, & \text{if } i \in D_j \text{ and } j = o_{t+1} \\ 0, & \text{otherwise} \end{cases}$$

If both $o_t$ and $o_{t+1}$ are unknown:

$$\xi_t(i,j) = \begin{cases} \frac{\alpha_t(i)a_{i,j}\beta_{t+1}(j)}{P(O|\lambda)}, & \text{if } i \in B_t \text{ and } j \in B_{t+1} \\ 0, & \text{otherwise} \end{cases}$$

Now to the updating of the transition probabilities. We intialise the algorithm with a uniform transition matrix, meaning each value in the matrix is $a_{i,j} = \frac{1}{\text{\# of states to go to from i}}$, if it is possible to go from $i$ to $j$, and 0 otherwise. We then need to calculate the $\xi$-probabilities for all of the given observation sequences. When we have the $\xi$-probabilities we can update our transition matrix. This is done the following way:

$$\hat{a}_{i,j} = \frac{\sum_{r=1}^{R}\sum_{t=1}^{T-1}\xi_{t,r}(i,j)}{\sum_{r=1}^{R}\sum_{k=1}^{N}\sum_{t=1}^{T-1}\xi_{t,r}(i,k)}, \tag{4}$$

where $\xi_{t,r}(i,j)$ is $\xi_t(i,j)$ for observation sequence $r$.

To illustrate the subsets of space involved in a calculation, Figure S2 shows all the nodes which will be given a value $> 0$ for (a) the $\alpha$-probabilities, (b) the $\beta$-probabilities, and (c) the $\xi$-probabilities for the observation 1010.

# A Hypercubic Baum-Welch example calculation

To illustrate the algorithm, we now consider an explicit example. Assume we are given 10100 as an observation in a cross-sectional context. That is interpreted as the sequence 00000 $\rightarrow$ ? $\rightarrow$ 10100 $\rightarrow$ ? $\rightarrow$ ? $\rightarrow$ 11111. We begin with a uniform transition matrix, and proceed to calculate the $\alpha$-, $\beta$-, and $\xi$-probabilities. Calculations are described one timepoint at a time in the text, and illustrated in Fig. S3.

## A.1 $\alpha$ calculations

We first need to calculate the forward probabilities, defined as follows:

$$\alpha_t(i) = P(o_0, ..., o_t = 1|\lambda)$$

For $t = 0$ we have $\alpha_0(00000) = 1$ and $\alpha_0(i) = 0$, for all $i$ except 00000. This will always be the case independent of the observation.

For $t = 1$ we will have $\alpha_1(i) = P(o_0 = 00000, o_1 = i) = P(00000 \to i)$. This will only have a value for $i \in \{10000, 01000, 00100, 00010, 00001\}$ since we are assuming that we only acquire one trait at a time. For the case where $i \in \{10000, 01000, 00100, 00010, 00001\}$ we have $\alpha_1(i) = P(00000 \to i) = a_{00000,i} = \frac{1}{5}$, and for everything else we have $\alpha_1(j) = 0$.

For $t = 2$ we have $\alpha_2(10100) = P(o_0 = 00000, o_1, o_2 = 10100) = \sum_j P(o_0 = 00000, o_1 = j)P(j \to 10100) = \sum_j \alpha_1(j) \cdot a_{j,10100}$. Here only $j \in \{10000, 00100\}$ give a non-zero value for both $\alpha_1(j)$ and $a_{j,10100}$. Hence, we end up with $\alpha_2(10100) = \alpha_1(10000)a_{10000,10100} + \alpha_1(00100)a_{00100,10100} = \frac{1}{5} \cdot \frac{1}{4} + \frac{1}{5} \cdot \frac{1}{4} = \frac{1}{10}$.

For $t = 3$ we have $\alpha_3(i) = P(o_0 = 00000, o_1, o_2 = 10100, o_3 = i) = P(o_0 = 00000, o_1, o_2 = 10100) \cdot a_{10100,i} = \alpha_2(10100) \cdot a_{10100,i}$. Here only the cases where it is possible to go from 10100 will have non-zero values. Hence, for $i \in \{11100, 10110, 10101\}$ we will have $\alpha_3(i) = \frac{1}{10} \cdot \frac{1}{3} = \frac{1}{30}$.

For $t = 4$ we have $\alpha_4(i) = P(o_0 = 00000, o_1, o_2 = 10100, o_3, o_4 = i) = \sum_j P(o_0 = 00000, o_1, o_2 = 10100, o_3 = j) \cdot a_{j,i} = \sum_j \alpha_3(j) \cdot a_{j,i}$. Here again we will only have a few possible non-zero values. Since $\alpha_3(j) \neq 0$ only for $j \in \{11100, 10110, 10101\}$ only the places we can go from these three will have non-zero values for $\alpha_4$. Hence, if $i \in \{11110, 11101, 10111\}$ then $\alpha_4(i) = \frac{1}{30} \cdot \frac{1}{2} + \frac{1}{30} \cdot \frac{1}{2} = \frac{1}{30}$. This is happening since for all $j \in \{11100, 10110, 10101\}$ there is only two possible ways to go.

For $t = 5$ we have $\alpha_5(11111) = P(o_0 = 00000, o_1, o_2 = 10100, o_3, o_4, o_5 = 11111) = \sum_j P(o_0 = 00000, o_1, o_2 = 10100, o_3, o_4 = j) \cdot a_{j,11111} = \sum_j \alpha_4(j) \cdot a_{j,11111} = \sum_j \alpha_4(j) = \frac{1}{30} + \frac{1}{30} + \frac{1}{30} = \frac{1}{10}$. For $t = 5$ everything else will be 0.

## A.2 $\beta$ calculations

$$\beta_t(i) = P(o_{t+1}, ..., o_T | o_t = i, \lambda)$$

For $t = 5$ we have $\beta_5(i) = 1$ for every $i$.

For $t = 4$ we have $\beta_4(i) = P(o_5 | o_4 = i) = P(11111 | o_4 = i) = a_{i,11111}$. If $i \in \{11110, 11101, 11011, 10111, 01111\}$ we will have $\beta_4(i) = 1$. For everything else $\beta_4(i) = 0$.

For $t = 3$ we have $\beta_3(i) = P(o_4, o_5 = 11111 | o_3 = i) = \sum_j P(o_5 = 11111 | o_4 = j) \cdot a_{i,j} \sum_j \beta_4(j) \cdot a_{i,j}$. From $t = 4$ we have that $\beta_4(j) = 1$ for $j \in \{11110, 11101, 11011, 10111, 01111\}$ and 0 otherwise. Hence we only need to calculate the values for every state where it is possible to reach one of these five values. Since these five values are all the possible states to be in at time $t = 4$ and the transition matrix is uniform $a_{i,j} = \frac{1}{2} > 0$ for every state that is possible to be in at time $t = 3$. We then end up with $\beta_3(i) = \frac{1}{2} + \frac{1}{2} = 1$ for all $i$ which is possible to be in at time $t = 3$.

For $t = 2$ we have $\beta_2(10100) = P(o_3, o_4, o_5 = 11111 | o_2 = 10100) = \sum_j P(o_4, o_5 = 11111 | o_3 = j) \cdot a_{10100,j} = \sum_j \beta_3(j) \cdot a_{10100,j}$. Here we can see that this will only have a non-zero value for states it is possible to go from 10100, and we know from time $t = 3$ that all non-zero values of $\beta_3$ is 1. Hence, we get $\beta_2(10100) = a_{10100,11100} \cdot a_{10100,10110} \cdot a_{10100,10101} = \frac{1}{3} \cdot \frac{1}{3} \cdot \frac{1}{3} = 1$.

For $t = 1$ we have $\beta_1(i) = P(o_2 = 10100, o_3, o_4, o_5 = 11111 | o_1 = i) = P(o_3, o_4, o_5 = 11111 | o_2 = 10100) \cdot a_{i,10100} = \beta_2(10100) \cdot a_{i,10100} = a_{i,10100}$. This will only have a non-zero value for states where it is possible to go to 10100. Hence, if $i \in \{10000, 00100\}$ we have $\beta_1(i) = a_{i,10100} = \frac{1}{4}$.

For $t = 0$ we have $\beta_0(00000) = P(o_1, o_2 = 10100, o_3, o_4, o_5 = 11111 | o_0 = 00000) = \sum_j P(o_2 = 10100, o_3, o_4, o_5 = 11111 | o_1 = j) \cdot a_{00000,j} = \sum_j \beta_1(j) \cdot a_{00000,j}$. We know from time $t = 1$ that $\beta_1(j) > 0$ only for $j \in \{10000, 00100\}$. Hence, we get $\beta_0(00000) = \beta_1(10000) \cdot a_{00000,10000} + \beta_1(00100) \cdot a_{00000,00100} = \frac{1}{4} \cdot \frac{1}{5} + \frac{1}{4} \cdot \frac{1}{5} = \frac{1}{10}$.

## A.3 $\xi$ calculations

$$\xi_t(i, j) = P(o_t = i, o_{t+1} = j) = \alpha_t(i) a_{i,j} \beta_{t+1}(j)$$

For $t = 0$ we have $\xi_0(00000, j) = P(o_0 = 00000, o_1 = j)$. This will only be non-zero for values where it is possible to go to from 00000. We also see that from the $\beta$-calculations that only the states 10000 and 00100 have non-zero values for $\beta_1$ so we only need to look at these two states. Again, since our transition matrix is uniform so every calculation will be similar. So, if $j \in \{10000, 00100\}$ we have $\xi_0(00000, j) = \alpha_0(00000) a_{00000,j} \beta_1(j) =$

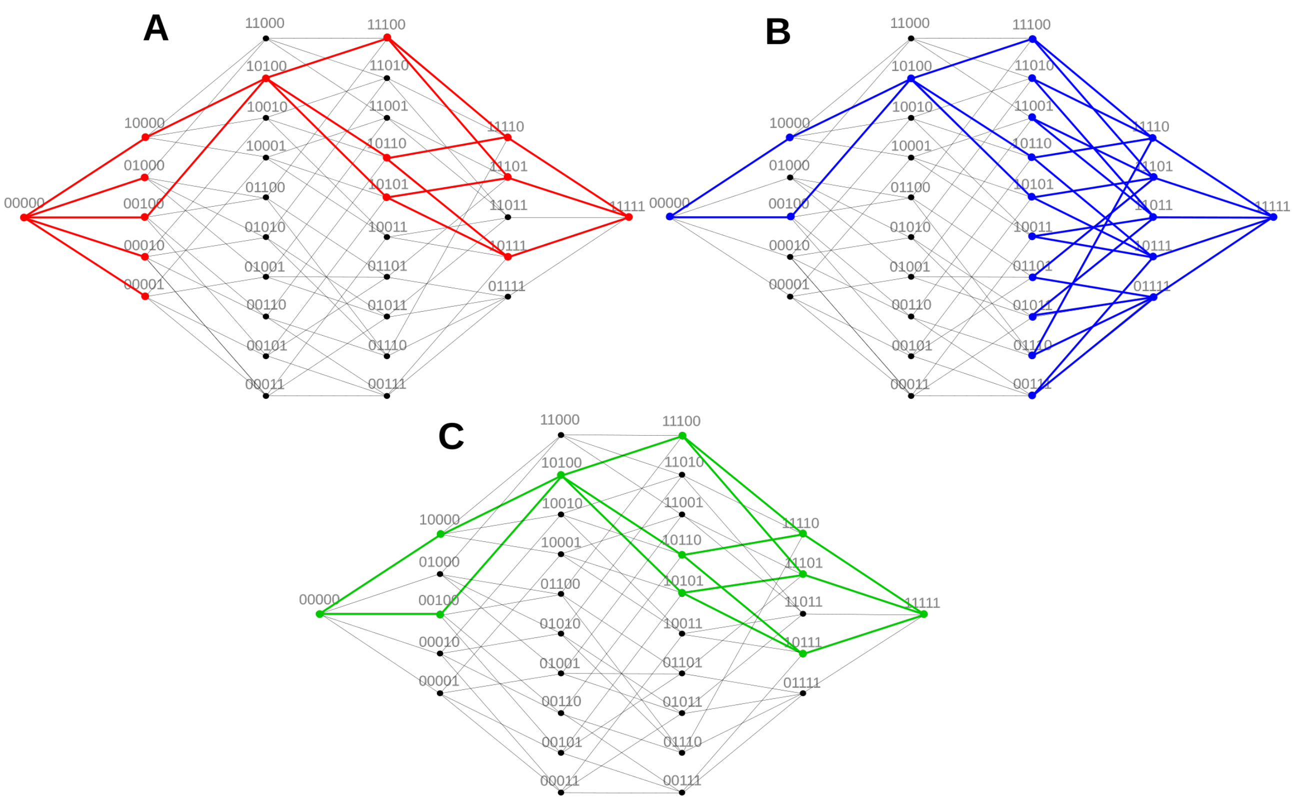


Figure S3: **Example edges considered in the HBW calculation.** Given the observation 10100, (A) shows all the nodes that will have $\alpha_t(i) > 0$ for any time $t$; (C) shows all the nodes that will have $\beta_t(i) > 0$ for any time $t$; (C) shows all the nodes that are included in both (A) and (B). All the edges connecting two nodes here will be given some weight when uploading the transition probabilities.

$1 \cdot \frac{1}{5} \cdot \frac{1}{4} = \frac{1}{20}$. For every other combination of $\xi_0(i,j) = 0$.

For $t = 1$ we have $\xi_1(i, 10100) = P(o_1 = i, o_2 = 10100) = \alpha_1(i) a_{i,10100} \beta_2(10100)$. From the $\alpha$-calculations we know that $\alpha_1(i) = \frac{1}{5}$ for $i \in \{10000, 01000, 00100, 00010, 00001\}$, but here we also see that only 10000 and 00100 will give a non-zero value for $a_{i,10100}$. Hence, we end up with $\xi_1(10000, 10100) = \frac{1}{5} \cdot \frac{1}{4} \cdot 1 = \frac{1}{20}$ and $\xi_1(00100, 10100) = \frac{1}{5} \cdot \frac{1}{4} \cdot 1 = \frac{1}{20}$.

For $t = 2$ we have $\xi_2(10100, j) = P(o_2 = 10100, o_3 = j) = \alpha_2(10100) a_{10100,j} \beta_3(j)$. Since all states which is possible to be in at time $t = 3$ have a value for $\beta_3$ we only need to consider the allowed transitions from 10100. Which gives us, for $j \in \{11100, 10110, 10101\}$ we have $\xi_2(10100, j) = \frac{1}{10} \cdot \frac{1}{3} \cdot 1 = \frac{1}{30}$.

For $t = 3$ we have $\xi_3(i,j) = P(o_3 = i, o_4 = j) = \alpha_3(i) a_{i,j} \beta_4(j)$. Again, from the $\alpha$-calculations we have that $\alpha_3(i) > 0$ for $i \in \{11100, 10110, 10101\}$. For $j$ we will have non-zero values when it is possible to go from on of the states $i$. So, if $i \in \{11100, 10110, 10101\}$ and $j \in \{11110, 10111, 11101\}$ we have $\xi_3(i,j) = \frac{1}{30} \cdot \frac{1}{2} \cdot 1 = \frac{1}{60}$.

For $t = 4$ we have $\xi_4(i, 11111) = P(o_4 = i, o_5 = 11111) = \alpha_4(i) a_{i,11111} \beta_5(11111)$. Here the calculations are restricted by the non-zero values of $\alpha_4(i)$. For $i \in \{11110, 10111, 11101\}$ we have $\xi_4(i, 11111) = \frac{1}{30} \cdot 1 \cdot 1 = \frac{1}{30}$.

# B Prior information and model selection

Although we cannot encode arbitrary prior information without a Bayesian picture, we can make some progress by encoding prior information in the structure of the hypercubic network that underlies our model. Say we know that trait $a$ always appears before trait $b$. A simple way of including this prior information is by removing all edges in the hypercubic transition network that correspond to an acquisition of $b$ from a state without trait $a$. The possibility of such a transition is thus removed from the inference process, and the HBW algorithm will identify the maximum likelihood parameterisation of the remaining edges given the observed data.

This approach of removing edges also allows a degree of model selection to be applied. We can consider different models for a given system, involving hypercubic networks with different edge sets removed. The HBW algorithm will identify a maximum likelihood parameterisation for each (or, if a chosen model is incompatible

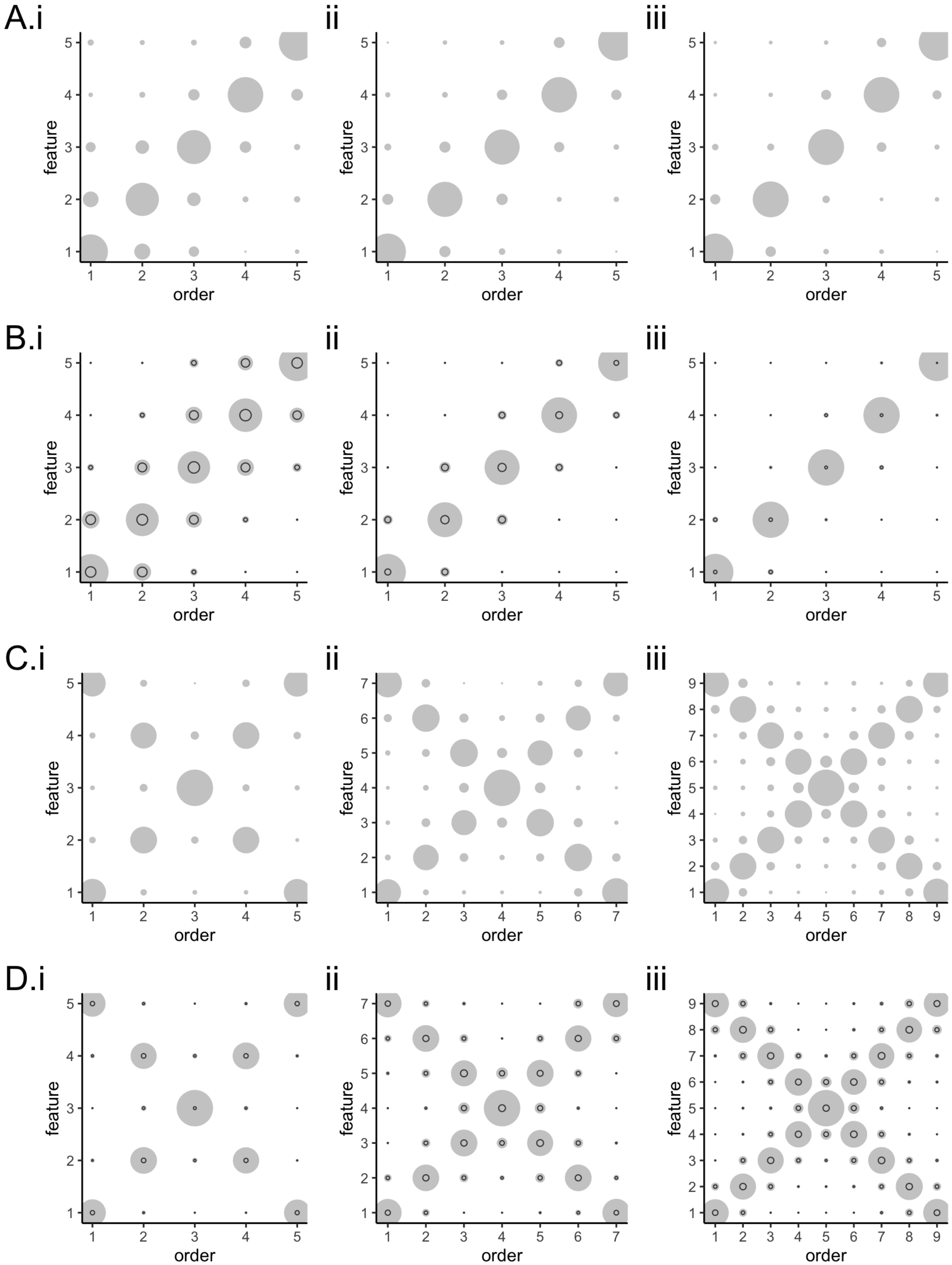


Figure S4: **Inferred dynamics for further synthetic test datasets.** (A-B) Single pathway with varying sample size using (A) HyperTraPS and (B) HyperHMM. (i) $n = 4$, (ii) $n = 8$, (iii) $n = 16$. (C-D) double pathway with varying feature count examples from the text using (C) HyperTraPS and (D) HyperHMM. (i) $L = 5$, (ii) $L = 7$, (iii) $L = 9$. Bubbles show the probability of getting trait $y$ at time $x$; black circles in the HyperHMM plots shows the standard deviation after 100 bootstraps.

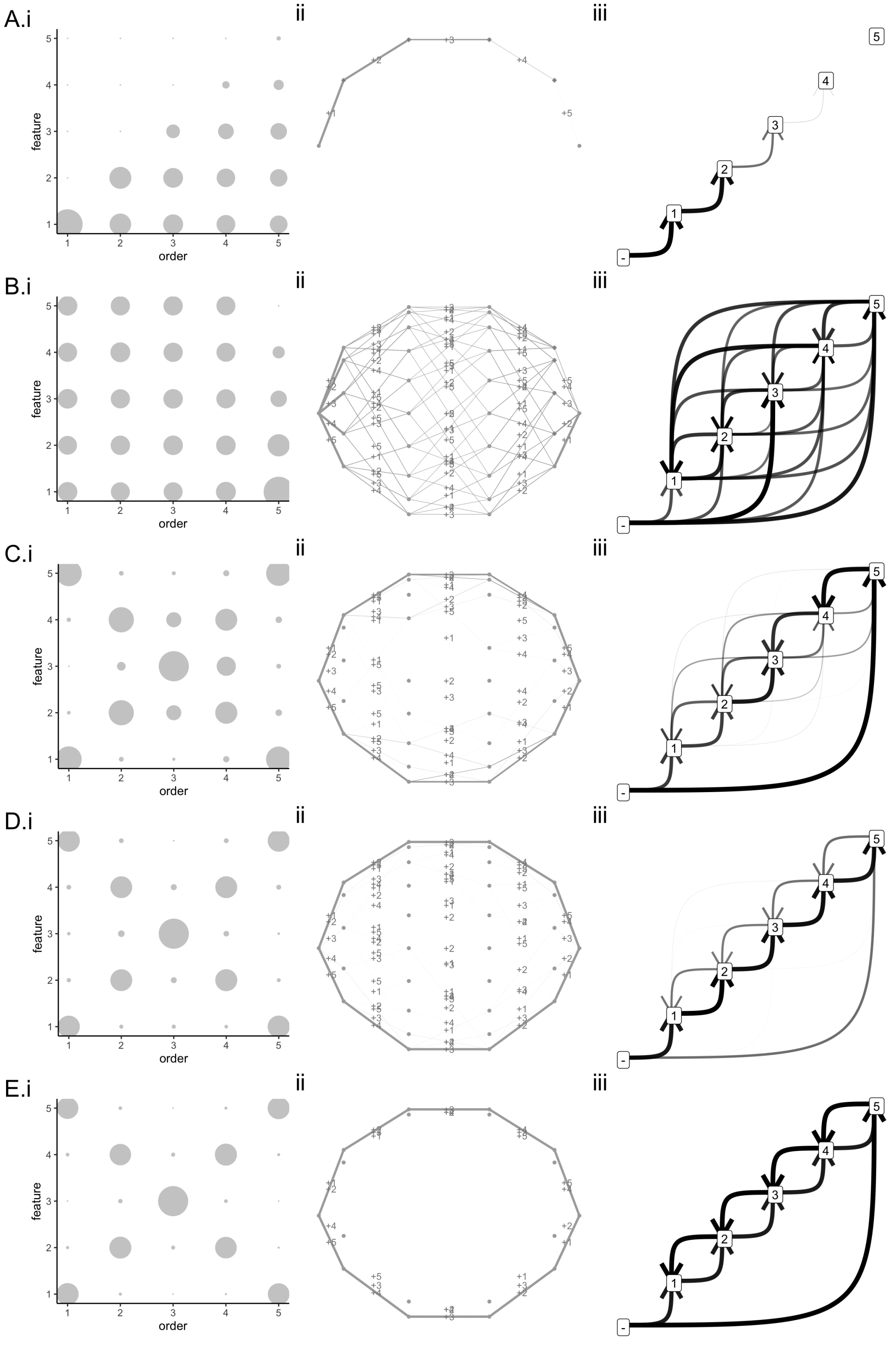


Figure S5: **Comparison of inference approaches for the synthetic double-pathway case.** (A) The logical relationships detected by Capri in the TRONCO system; simulated dynamics from (B) Oncotree; (C) Mutual Hazard Networks; (D) HyperTraPS using an $L^2$ parameterisation; (E) HyperHMM. (i) Bubble plot summaries of mean feature orderings; (ii) inferred paths on the hypercubic transition network; (iii) inferred ordered pairs of acquisitions. Here, the mutually-repressing nature of the paths is captured by the approaches that allow pairwise negative influences between traits (C-E).

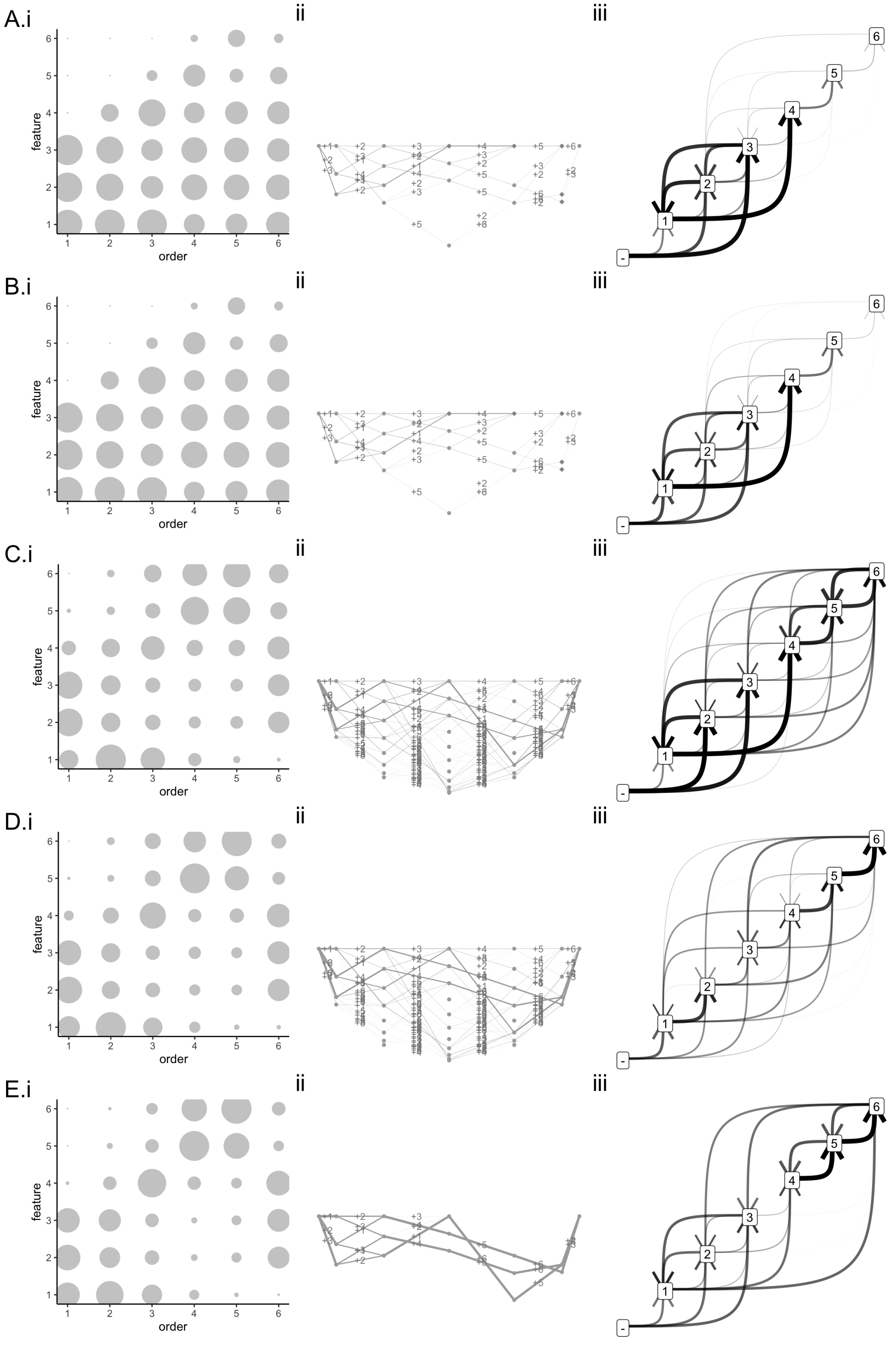


Figure S6: **Comparison of inference approaches for the synthetic higher-order influence case.** (A) The logical relationships detected by Capri in the TRONCO system; simulated dynamics from (B) Oncotree; (C) Mutual Hazard Networks; (D) HyperTraPS using an $L^2$ parameterisation; (E) HyperHMM. (i) Bubble plot summaries of mean feature orderings; (ii) inferred paths on the hypercubic transition network; (iii) inferred ordered pairs of acquisitions. Here, various approaches approximate the system's behaviour, but those allowing only pairwise interactions (C-D) include several spurious transitions and do not capture the full logical dependence. Only (E), allowing arbitrary-order interactions, captures these influences precisely.

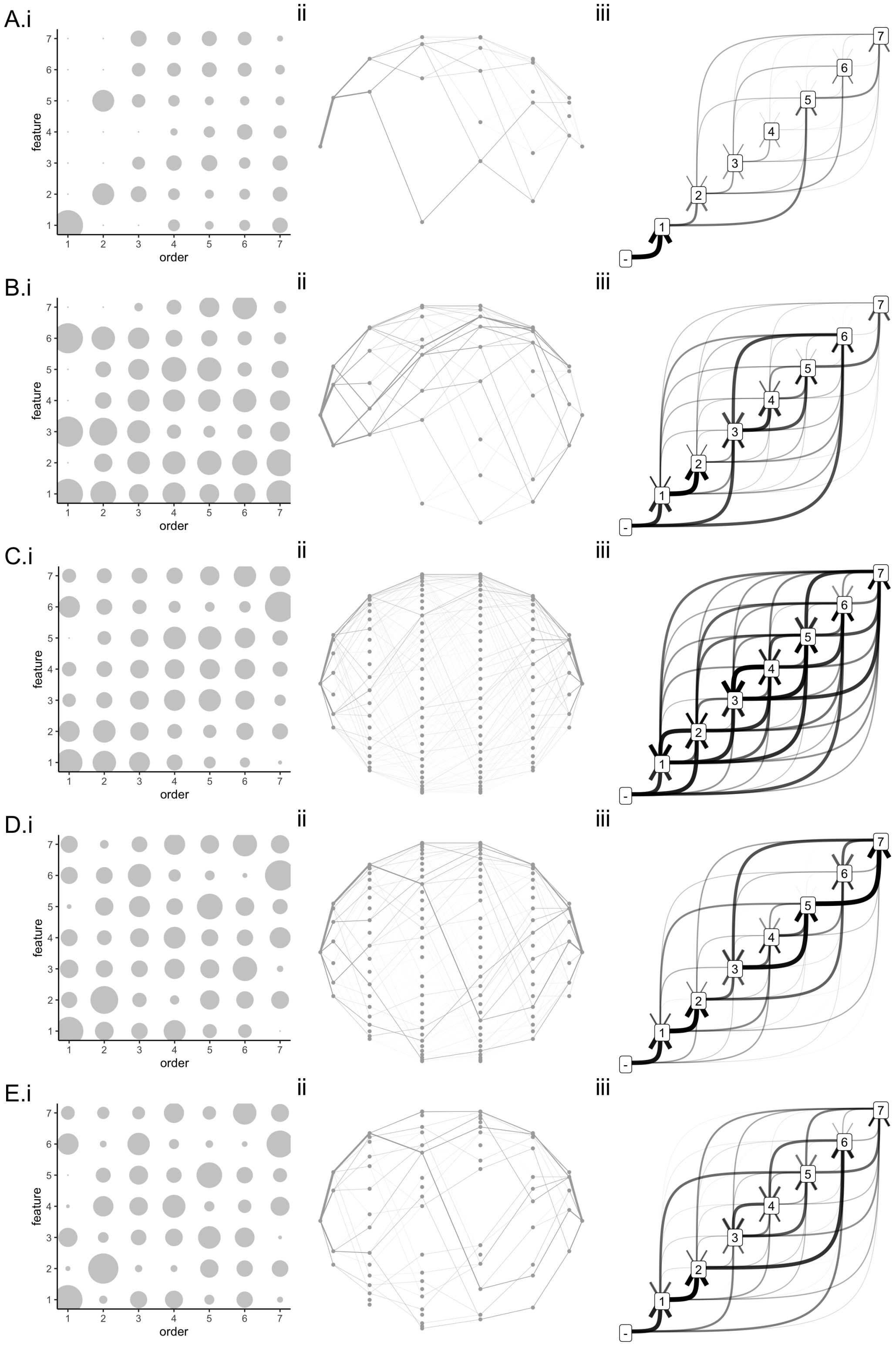


Figure S7: **Comparison of inference approaches for the ovarian cancer case.** (A) The logical relationships detected by Capri in the TRONCO system; simulated dynamics from (B) Oncotree; (C) Mutual Hazard Networks; (D) HyperTraPS using an $L^2$ parameterisation; (E) HyperHMM. (i) Bubble plot summaries of mean feature orderings; (ii) inferred paths on the hypercubic transition network; (iii) inferred ordered paairs of acquisitions. Here, HyperHMM shares similar initial steps to the Oncotree picture, but thereafter shares more similarities with HyperTraPS in hypercubic path structure.

| Convergence time / s | | | |
|---|---|---|---|
| Dataset | Hypercubic Baum-Welch ($\epsilon = 0.001$) | HyperTraPS ($\epsilon$ = 0.001) | HyperTraPS ($\epsilon$ = 0.01) |
| $L = 5$ single | 0.0960 | 961 | 168 |
| $L = 7$ single | 0.768 | 3380 | 950 |
| $L = 9$ single | 3.70 | 8900 | 1308 |
| $L = 5$ double | 0.239 | 1181 | 305 |
| $L = 7$ double | 1.55 | 5914 | 1081 |
| $L = 9$ double | 6.06 | 16978 | 2521 |
| Ovarian | 19.3 | $> 2 \times 10^5$ | $> 2 \times 10^5$ |
| TB drug | 3480 | $\gg 2 \times 10^5$ | $> 2 \times 10^5$ |

Table S1: **Convergence times for different hypercubic inference approaches.** Timing results for various datasets using the HyperHMM algorithm and HyperTraPS. All times are in seconds; convergence criteria $\epsilon$ are the maximum change to any probability in the model allowed between instances (HBW iterations; HyperTraPS sample blocks). The HyperHMM column shows the time it took to run 100 bootstraps with a convergence criterion of 0.001.

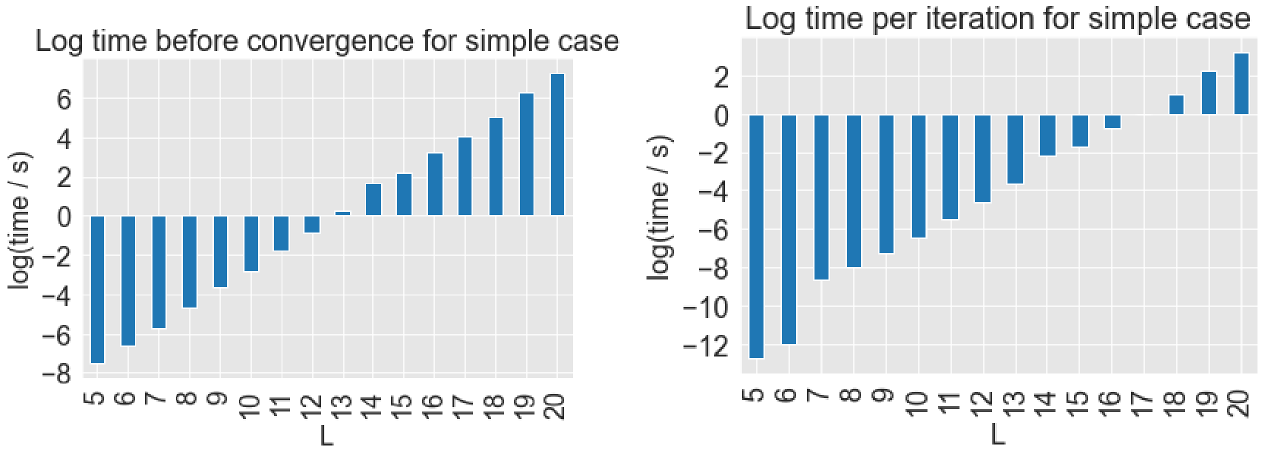


Figure S8: **Convergence behaviour with $L$.** Time per Baum-Welch iteration, and time to converged inference, for the simple (single pathway) synthetic dataset in the text with different $L$.

| | (a) full hypercube $k = 49$ | (b) single pathway $k = 0$ | (c) double pathway $k = 1$ |
|---|---|---|---|
| (i) single pathway | $\mathcal{L} \simeq 0.98$; $AIC \simeq 98$ | $\mathcal{L} = 1$; $AIC = 0$ | $\mathcal{L} = 1$; $AIC = 2$ |
| (ii) double pathway | $\mathcal{L} \simeq 1.45 \times 10^{-5}$; $AIC \simeq 120$ | $\mathcal{L} = 0$; $AIC = \infty$ | $\mathcal{L} = 0.5^{16} = 1.52 \times 10^{-5}$; $AIC \simeq 24$ |

Table S2: **Model selection for different $L = 5$ experiments.** We use the Akaike Information Criterion $AIC = 2k - 2\log\mathcal{L}$, where $\mathcal{L}$ is likelihood and $k$ is number of parameters. Rows give different synthetic datasets; columns give different parameter structures for the hypercubic model. In each case the most parsimonious model capable of capturing the data is favoured.

with observations, fail to identify any parameterisation and give a zero likelihood). The number of free parameters associated with a transition graph $G$ is the number of outgoing edges $d_{out}(s)$ from each non-terminal node $s$ minus one, summed over all nodes: $k(G) = \sum_{s \in G, d_{out}(s) > 0} (d_{out}(s) - 1)$. $k(G)$ for the full hypercube can readily be computed as $\sum_{n=0}^{L-1} \binom{L}{n}(L - n - 1) = 1 - 2^L + 2^{L-1}L$. Given this parameter count and the associated likelihood, we can then use model selection approaches like the Akaike and Bayesian Information Criteria to choose parsimonious hypercube structures that are compatible with observations. As a simple example, consider the single and double pathway models above. We can consider three different model structures: (a) the full hypercube (as in all previous sections); (b) the hypercube with all edges removed except those on the single pathway 000... → 100... → 110... → ...; (c) the hypercube with all edges removed except those on the two pathways 000... → ...001 → ...011 → .... Then consider finding the maximum likelihood parameterisations of each of these models with (i) $N$ samples from the single pathway system and (ii) $N$ samples from the double pathway system. The results, along with associated AIC values, are given in Table. S2, demonstrating that models that are unnecessarily complex (a) or insufficiently complex (b)(ii) are penalised in favour of those that better match the observational structure (b)(i), (c)(ii).

The principle of edge removal can also be used to regularise identified models, as demonstrated in Ref. [5]. Here, the full hypercube model is first parameterised, then those edges with the lowest weights are removed and a model selection criterion recalculated. The process continues to iteratively remove low-probability edges until an unacceptable likelihood penalty results. While not flawless (for example, the best solution might involve removing a combination of edges that is not encountered in the iterative one-at-a-time approach), this approach can be used to lower the complexity of learned models given a set of observations.

## C Library credits

The inference code uses the C++ Armadillo library [6]. Visualisations and wrapper scripts use R libraries stringr [7], ggplot2 [8], ggrepel [9], gridExtra [10], and igraph [11].

## References


[1] Sheldon M Ross. *Introduction to probability models.* Academic press, 2014.

[2] James H. Martin Dan Jurafsky. Speech and language processing, [online draft] (Appendix A), retrieved from https://web.stanford.edu/ jurafsky/slp3/ on 04/05/2021.

[3] Fanny Yang, Sivaraman Balakrishnan, and Martin J Wainwright. Statistical and computational guarantees for the Baum-Welch algorithm. *The Journal of Machine Learning Research*, 18(1):4528–4580, 2017.

[4] Lawrence R Rabiner. A tutorial on hidden Markov models and selected applications in speech recognition. *Proceedings of the IEEE*, 77(2):257–286, 1989.

[5] Sam F Greenbury, Mauricio Barahona, and Iain G Johnston. Hypertraps: Inferring probabilistic patterns of trait acquisition in evolutionary and disease progression pathways. *Cell systems*, 10(1):39–51, 2020.

[6] Conrad Sanderson and Ryan Curtin. Armadillo: a template-based c++ library for linear algebra. *Journal of Open Source Software*, 1(2):26, 2016.

[7] Hadley Wickham. *stringr: Simple, Consistent Wrappers for Common String Operations*, 2019. R package version 1.4.0.

[8] Hadley Wickham. *ggplot2: Elegant Graphics for Data Analysis.* Springer-Verlag New York, 2016.

[9] Kamil Slowikowski. *ggrepel: Automatically Position Non-Overlapping Text Labels with 'ggplot2'*, 2021. R package version 0.9.1.

[10] Baptiste Auguie. *gridExtra: Miscellaneous Functions for "Grid" Graphics*, 2017. R package version 2.3.

[11] Gabor Csardi and Tamas Nepusz. The igraph software package for complex network research. *InterJournal*, Complex Systems:1695, 2006.
